# Butyrate induced Tregs are capable of migration from the GALT to the pancreas to restore immunological tolerance during type-1 diabetes

**DOI:** 10.1038/s41598-020-76109-y

**Published:** 2020-11-05

**Authors:** Neenu Jacob, Shivani Jaiswal, Deep Maheshwari, Nayudu Nallabelli, Neeraj Khatri, Alka Bhatia, Amanjit Bal, Vivek Malik, Savita Verma, Rakesh Kumar, Naresh Sachdeva

**Affiliations:** 1 Department of Pediatrics, Post Graduate Institute of Medical Education and Research (PGIMER), Chandigarh, India; 2 Department of Endocrinology, Post Graduate Institute of Medical Education and Research (PGIMER), Chandigarh, India; 3 iCARE, Institute of Microbial Technology (IMTech), Chandigarh, India; 4 Department of Experimental Medicine and Biotechnology, Post Graduate Institute of Medical Education and Research (PGIMER), Chandigarh, India; 5 Department of Histopathology, Post Graduate Institute of Medical Education and Research (PGIMER), Chandigarh, India; 6 School of Public Health, Post Graduate Institute of Medical Education and Research (PGIMER), Chandigarh, India

**Keywords:** Immune tolerance, Chemokines, CD4-positive T cells, Type 1 diabetes, Dietary carbohydrates

## Abstract

Type-1 diabetes (T1D) is an autoimmune disease caused by progressive loss of insulin-producing beta cells in the pancreas. Butyrate is a commensal microbial-derived metabolite, implicated in intestinal homeostasis and immune regulation. Here, we investigated the mechanism of diabetes remission in non-obese diabetic (NOD) mice following butyrate administration. Sodium butyrate (150 mM) was administered to female NOD mice in drinking water after the onset of hyperglycemia (15–25 weeks age) and at 4 weeks of age (early-intervention group). Butyrate administration reduced the progression of hyperglycemia in diabetic mice and delayed onset of diabetes in the early-intervention group with a reduction in insulitis. Butyrate administration increased regulatory T cells (Tregs) in the colon, mesenteric lymph nodes, Peyer’s patches, and its protective effects diminished upon depletion of Tregs. Further, an increase in α4β7, CCR9, and GPR15 expressing Tregs in the pancreatic lymph nodes (PLN) and pancreas in butyrate-treated mice suggested migration of gut-primed Tregs towards the pancreas. Finally, the adoptive transfer experiments demonstrated that induced Tregs from gut-associated lymphoid tissue can migrate towards the pancreas and PLN and delay the onset of diabetes. Our results thus suggest that early administration of butyrate can restore immunological tolerance during T1D via induction of Tregs with migratory capabilities.

## Introduction

Type-1 diabetes (T1D) is a chronic autoimmune disease associated with the destruction of pancreatic beta cells, mediated mainly by the islet-reactive T cells. Short-chain fatty acid (SCFA)-producing bacteria are important determinants of gut homeostasis and implicated in the development of T1D^1^. SCFA includes mainly acetate, propionate, and butyrate which are metabolites produced by the microbial fermentation of dietary fiber and complex carbohydrates^2^. Among these SCFA, butyrate plays an important role in maintaining gut integrity by inducing synthesis of mucin and enhancing the production of tight junction proteins^3^. Butyrate can also induce functional colonic regulatory T cells (cTregs) by interacting with their specific receptors GPR43 or by inhibiting the histone deacetylases (HDAC) at the Forkhead box P3 (*FoxP3)* gene locus or via epigenetic upregulation of *Foxp3* gene^4,5^. Healthy children reported having an abundance of butyrate-producing bacteria in their microbiome compared to children having positivity for at least one islet autoantibody^6^. Reduced number of FoxP3 + Tregs due to their defective differentiation in the gut has been observed in T1D patients^7^.

Besides induction, variations in the expression of chemokines and their ligands in T cells including, Tregs have been demonstrated in NOD mice and T1D patients^8^. In particular, the CXCL12-CXCR4 pathway has been implicated in the retention of Tregs in the pancreatic lymph nodes (PLN) as well as beta cell regeneration in the NOD mice^9^. The PLNs, as well as mesenteric lymph nodes (MLN), drain the pancreatic tissue, establishing an immunologic connection between the gut-associated lymphoid tissue (GALT) and the pancreatic islets. This connection is evident from the preferential trafficking of T cells from gut to PLN, where they can be activated by the antigens draining from the gastrointestinal tract^10^. Also, the T cells activated in the gastrointestinal tract can migrate to pancreatic islets due to the expression of MAdCAM-1^11,12^. Previous reports have demonstrated that T cells migrating from the intestinal mucosa to the PLN express gut-homing receptors such as α4b7 and CCR9 in the pancreatic islets of NOD mice^13^ as well as in the T1D patients^14^. Another chemoattractant receptor, GPR15, has also been shown to specifically support preferential trafficking of Tregs towards the gut^15^, providing evidence of marking Tregs originating in the gut. Thus, the Tregs generated in the gut can strike the imbalance between effector T cells (Teff) cells and Tregs in the pancreas and suppress the Teff cell responses in the pancreas and PLN.

While studies have shown that butyrate can induce and expand cTregs, there is little knowledge about whether these cTregs can modulate islet-associated autoimmune responses in T1D. In this study, we evaluated the induction of cTregs as well as Tregs in other GALT after oral administration of sodium butyrate and assessed its effect on the remission of diabetes in NOD mice. We analyzed the migratory ability of these induced Tregs to the pancreas and PLN, to further understand their role in the suppression of diabetes in NOD mice.

## Results

### Sodium butyrate treatment delays progression of hyperglycemia in diabetic NOD mice

Before starting the experiments, we assessed whether addition of 150 mM sodium butyrate affected water consumption in mice until 21 days. No difference in the volume of water consumed by butyrate treated and water control hyperglycemic NOD mice was observed during the observation period (Supplementary Table S1).

**Figure 1:**
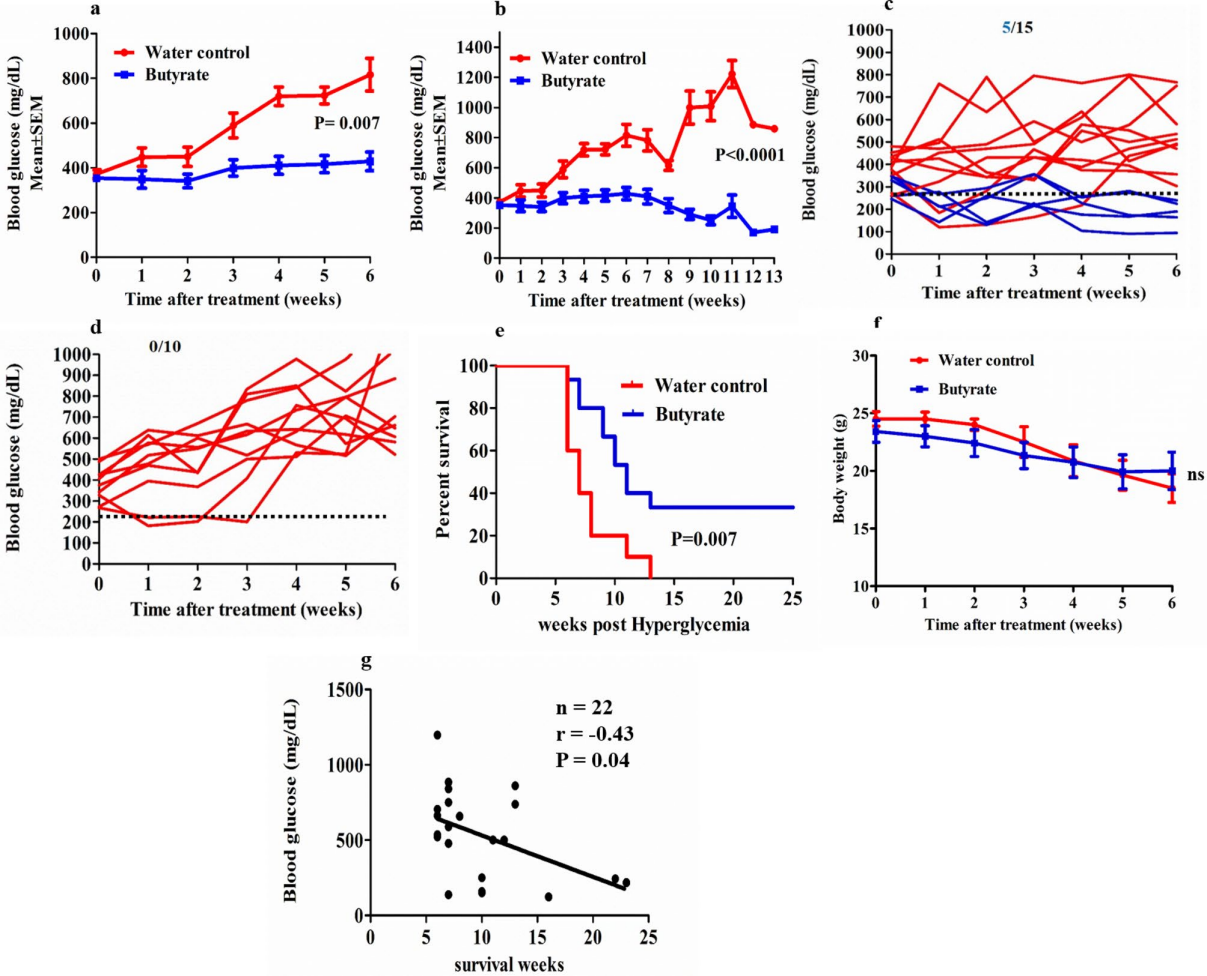
Sodium butyrate treatment delays progression of hyperglycemia in diabetic NOD mice. Hyperglycemic NOD mice (age 15–25 weeks) were given sterile drinking water with or without sodium butyrate (150 mM) until the maximum survival time or for 25 weeks post hyperglycemia. (**a**) The trend in the blood glucose levels for 6 weeks post hyperglycemia, each data point represents mean ± SEM of blood glucose of treatment group (n = 15) and control group (n = 10). (**b**) The trend in the blood glucose levels up to 13 weeks post hyperglycemia, each data point represents mean ± SEM of blood glucose of treatment group (n = 15) and control group (n = 10).The blood glucose levels of individual mice in (**c**) treatment (n = 15) and (**d**) control group (n = 10) up to 6 weeks post hyperglycemia. The dotted line represents blood glucose level at 250 mg/dL. (**e**) The percent survival of hyperglycemic NOD mice, treatment group (n = 15) and control group (n = 10) (Kaplan Meier log-rank test). (**f**) Body weight (in grams) of NOD mice upto 6 weeks post hyperglycemia in treatment group (n = 15) and control group (n = 10). (**g**) Correlation between blood glucose levels (measured at the maximum survival time period or up to 25 weeks post-hyperglycemia in NOD mice showing diabetes remission) and maximum survival time period (weeks) (n = 22) was performed with Spearman’s rank correlation test. Statistical significance was determined by Mann–Whitney U test, with P < 0.05 considered as significant; ns, not significant. Data are shown as mean ± SEM.

At first, we evaluated the therapeutic effect of sodium butyrate in NOD mice after the onset of diabetes. The blood glucose levels (mg/dL) were measured every week from 1st week onwards following butyrate administration. We observed a significant decrease in the blood glucose levels in the butyrate treatment group (385.4 ± 13.8, mean ± SEM) compared to the control group (562.2 ± 55.2, mean ± SEM) over the period of six weeks of treatment (P = 0.007) (Fig. 1a). Beyond 6 weeks, death was observed in the control group. However, further analysis of the blood glucose levels until 13 weeks post hyperglycemia (which was the maximum time point of survival of the control group) also showed a significant difference in the blood glucose levels in the treatment group compared to the control group (336.2 ± 22 vs 749.3 ± 64.6, mean ± SEM) (P < 0.0001) (Fig. 1b). We also assessed the reversal of diabetes in these hyperglycemic groups. Post-six weeks treatment, 5 out of 15 mice in the butyrate treatment group (33%) showed complete diabetes reversal (blood glucose < 250 mg/dL) (Fig. 1c), while all control groups remained hyperglycemic (0%) (Fig. 1d).

Further, we evaluated the effects of butyrate treatment on the percentage of survival of NOD mice. Post hyperglycemia, the median survival time was 11 weeks in the treatment group and 7 weeks in the control group, (P = 0.007) (Fig. 1e). The maximum survival time for the control group was 13 weeks, while 5 out of 15 mice in the treatment group survived for more than 25 weeks post hyperglycemia (Fig. 1e). Upon assessment of body weight, there was a trend in the reduction of body weight for 6 weeks post hyperglycemia, although overall there was no significant difference between the treatment and the control groups (P = 0.61) (Fig. 1f). To investigate whether the blood glucose levels had any impact on the survival of NOD mice, we analyzed the correlation between blood glucose levels and the maximum survival time. We found a significant negative correlation between the blood glucose levels and weeks survived by the mice (r = − 0.43, P = 0.04) (Fig. 1g). These observations suggest that sodium butyrate administration was able to control hyperglycemia as well as improve the survival of diabetic NOD mice.

### Butyrate treatment causes a reduction in insulitis and beta cell destruction

**Figure 2:**
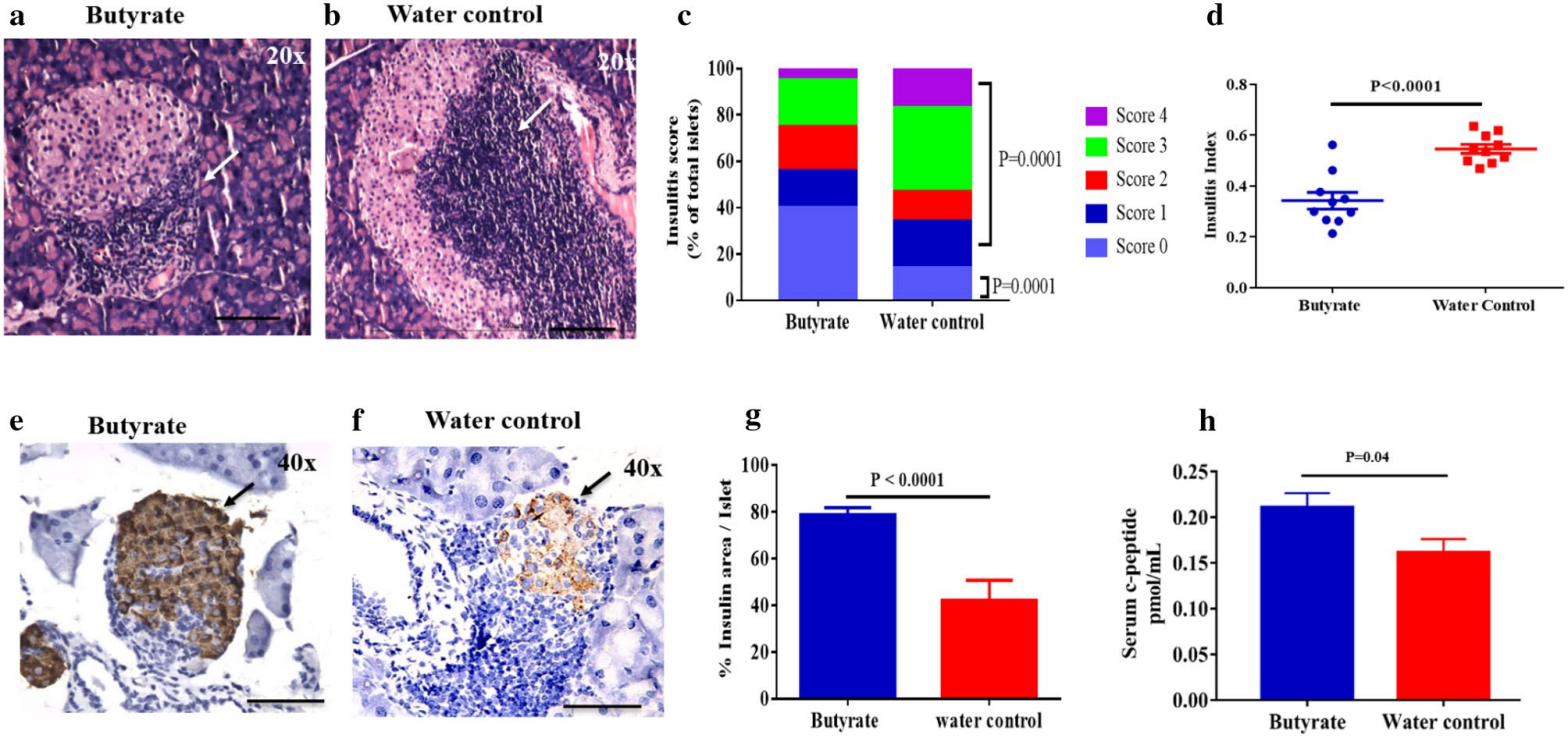
Butyrate treatment reduces insulitis and preserves insulin content of the hyperglycemic NOD mice pancreas. Hyperglycemic NOD mice (age 15–25 weeks) were given sterile drinking water with or without sodium butyrate (150 mM) for six weeks. Insulitis was determined by haematoxylin and eosin staining of the pancreatic sections at six weeks post-treatment. Representative images showing immune cell infiltration in (**a**) Butyrate-treated and, (**b**) water control groups. Arrows show region of infiltration at 20 × magnification; scale bars indicate 200 µm. (**c**) The degree of insulitis was scored as percent of islets with score 0, score 1, score 2, score 3 and score 4 from butyrate treated (n = 9) and control mice (n = 9). Insulitis between treatment and control groups were compared using Fisher’s exact test. Minimum of 20 islets from each mice were evaluated for insulitis. (**d**) The insulitis index was calculated according to the formula: Insulitis index = (0 × n_0_) + (1 × n_1_) + (2 × n_2_) + (3 × n_3_) + (4 × n_4_) / 4 (n_0_ + n_1_ + n_2_ + n_3_ + n_4_). Insulin staining was determined by immunohistochemistry of the pancreatic sections at six weeks post treatment. Representative histological section of NOD mice pancreas showing insulin stained islets in (**e**) Butyrate treated hyperglycemic NOD mice and, (**f**) Untreated hyperglycemic NOD mice. Arrows show region of infiltration at 40 × magnification; scale bars indicate 100 µm. (**g**) Immunohistochemical analysis of the insulin stained pancreatic islets from butyrate-treated (n = 5) or control (n = 4) NOD mice was determined by the percentage ratio of insulin-cell stained area to the total area of the same islet using Image J software. More than 50 islets from each group were evaluated for insulin staining. Post-six weeks treatment of hyperglycemic NOD mice, (**h**) serum C-peptide levels from treatment (n = 10) and control (n = 14) mice were determined by ELISA. Data are shown as mean ± SEM. Statistical significance between treatment and control groups was determined by Mann–Whitney U test, with P < 0.05 considered as significant.

To assess if butyrate treatment had any effect on reducing the immune cell infiltration into the pancreatic islets, 15–25 weeks old hyperglycemic NOD female mice were treated with sodium butyrate along with their respective controls. Insulitis scoring of the NOD mice was performed (Supplementary Fig S1) and the degree of insulitis was determined in treated and control mice (Fig. 2a–c). The treated mice showed larger islet regions that were free of lymphocytic infiltration (score 0, 40%) compared to the control mice (score 0, 14%) (P = 0.0001) (Fig. 2c). Peri-insulitis (score 1) was observed in 16.2% of islets in the treated group and 20.3% of islets in the control group (P = 0.6). Overall, the treated mice displayed reduction in insulitis (combined scores 1–4) compared to the control group (60% vs 86%, P = 0.0001) (Fig. 2c), with a significant reduction in total insulitis index (P < 0.0001) (Fig. 2d).

Further, the pancreas were histologically analyzed for insulin content to determine the functional beta cell reserve. The treatment group showed a lesser loss of insulin-positive cells (Fig. 2e) compared to the control groups (Fig. 2f). The histomorphometric quantification of insulin-positive islets confirmed this observation with butyrate treated group showing 78.7 (± 3.2)% insulin-positive islets, while the control group showed 41.9 (± 8.8)% insulin-positive islets (P < 0.0001) (Fig. 2g). In addition, we observed that serum C-peptide levels in the treatment group were also higher (P = 0.04) (Fig. 2h).

Overall, these results suggest that butyrate treatment is able to limit insulitis and preserve functional beta cell mass.

### Sodium butyrate delays the onset of diabetes in the early-intervention group

**Figure 3:**
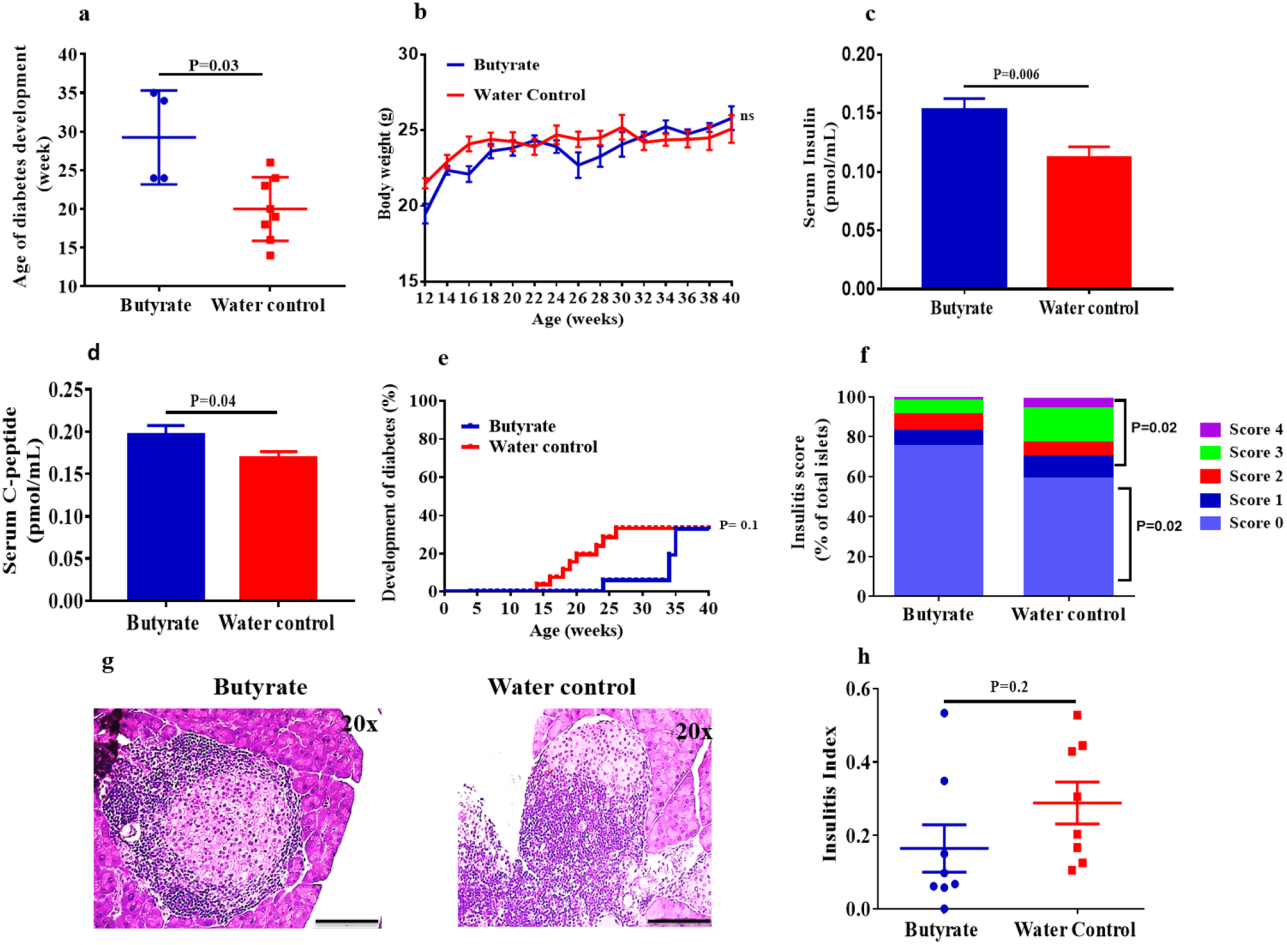
Sodium butyrate delays the incidence of diabetes in the early-intervention group. NOD mice (age 4 weeks) were treated with sodium butyrate (150 mM) or with sterile water until 40 weeks of age. (**a**) Age of onset of diabetes in treatment (n = 4) and control (n = 8) groups, (**b**) Body weight (in grams) of treatment (n = 20) and control (n = 17) groups at different time points up to 40 weeks of age; each data point represents (mean ± SEM). (**c**) Serum insulin (pmol/mL) levels and (**d**) Serum C-peptide (pmol/mL) levels in the treatment (n = 8) and the control groups (n = 8) was analyzed by ELISA from 30-week old NOD mice. (**e**) The development of diabetes in the treatment (n = 20) and the control (n = 17) groups at different time points up to 40 weeks of age (Kaplan Meier log-rank test). (**f**) Insulitis was scored as percent of islets with score 0, score 1, score 2, score 3 and score 4 from butyrate treated (n = 8) and control mice (n = 8) at 8 weeks of age. Insulitis between treatment and control groups were compared using Fisher’s exact test. Minimum of 20 islets from each mice in butyrate treated and control groups were evaluated for insulitis. (**g**) Representative images of the immune cell infiltration in butyrate-treated and control groups from 30 weeks old NOD mice at 20 × magnification, scale bars indicate 200 µm. (**h**) The insulitis index was obtained according to the formula: Insulitis index = (0 × n_0_) + (1 × n_1_) + (2 × n_2_) + (3 × n_3_) + (4 × n_4_) / 4 (n_0_ + n_1_ + n_2_ + n_3_ + n_4_). Statistical significance was determined by Mann–Whitney U test, with P < 0.05 considered as significant; ns, not significant. Data are shown as mean ± SEM.

Since the clinical symptoms of T1D are preceded by beta cell autoimmunity, we speculated that 4 weeks would be a good starting time for the therapeutic intervention in NOD mice. Hence, we assessed the effect of sodium butyrate in the NOD mice by first investigating the incidence of diabetes onset. We observed a significant delay in the mean age of onset of diabetes in the early-intervention group (29.3 ± 3 weeks) in comparison to the control group (20.0 ± 1.5 weeks) (P = 0.03) (Fig. 3a). There was no significant difference in the bodyweight of early-intervention versus control groups at different time points (Fig. 3b). Upon evaluation of the random serum C-peptide and insulin levels at 30 weeks of age, we observed higher insulin (P = 0.006) (Fig. 3c) and C-peptide (P = 0.04) (Fig. 3d) levels in the early-intervention groups. By the end of 40 weeks age, 4 out of 20 (20%) mice in the early-intervention group developed diabetes as compared to 8 out of 17 (47%) mice in the control group (P = 0.1) (Fig. 3e), indicating that the protective effect of butyrate is gradually lost with time as the disease progresses. Further, we assessed insulitis in the pre-diabetic stage. At 8 weeks of age, the treated mice showed larger islet regions that were free of lymphocytic infiltration (75%) compared to the control mice (59%) (P = 0.02, Score 0) (Fig. 3f). Peri-insulitis was observed in 8.4% of the islets in the treated and 11.2% of the islets in the control group (P = 0.6). Overall, there was a reduction in insulitis in the treated mice (combined scores 1–4) compared to the control group (25% vs 41%, P = 0.02) (Fig. 3f,g). However, we did not observe any reduction in the total insulitis index in the treatment group (P = 0.2) (Fig. 3h).

### Butyrate treatment induces Tregs in the colon and the GALT

In order to assess that sodium butyrate administration resulted in the relevant levels of butyrate in the colon, we performed GC–MS analysis of the colonic extracts of NOD mice. The presence of butyrate was confirmed from the NIST library. The qualitative GC–MS analysis of colonic contents demonstrated the presence of butyric acid (m/z = 145.1) in the butyrate treated and water control mice (Supplementary Fig S2a). Trace amounts of butyrate were also observed from the colonic extracts of the vancomycin treated mice that received plain drinking water. The peak area under curve (AUC) indicating butyrate content in the colonic extract of the butyrate treated mice was much higher compared to the control mice and vancomycin treated mice (Supplementary Fig S2c,d,e), suggesting that oral butyrate administration results in higher levels of butyrate in the colon.

**Figure 4:**
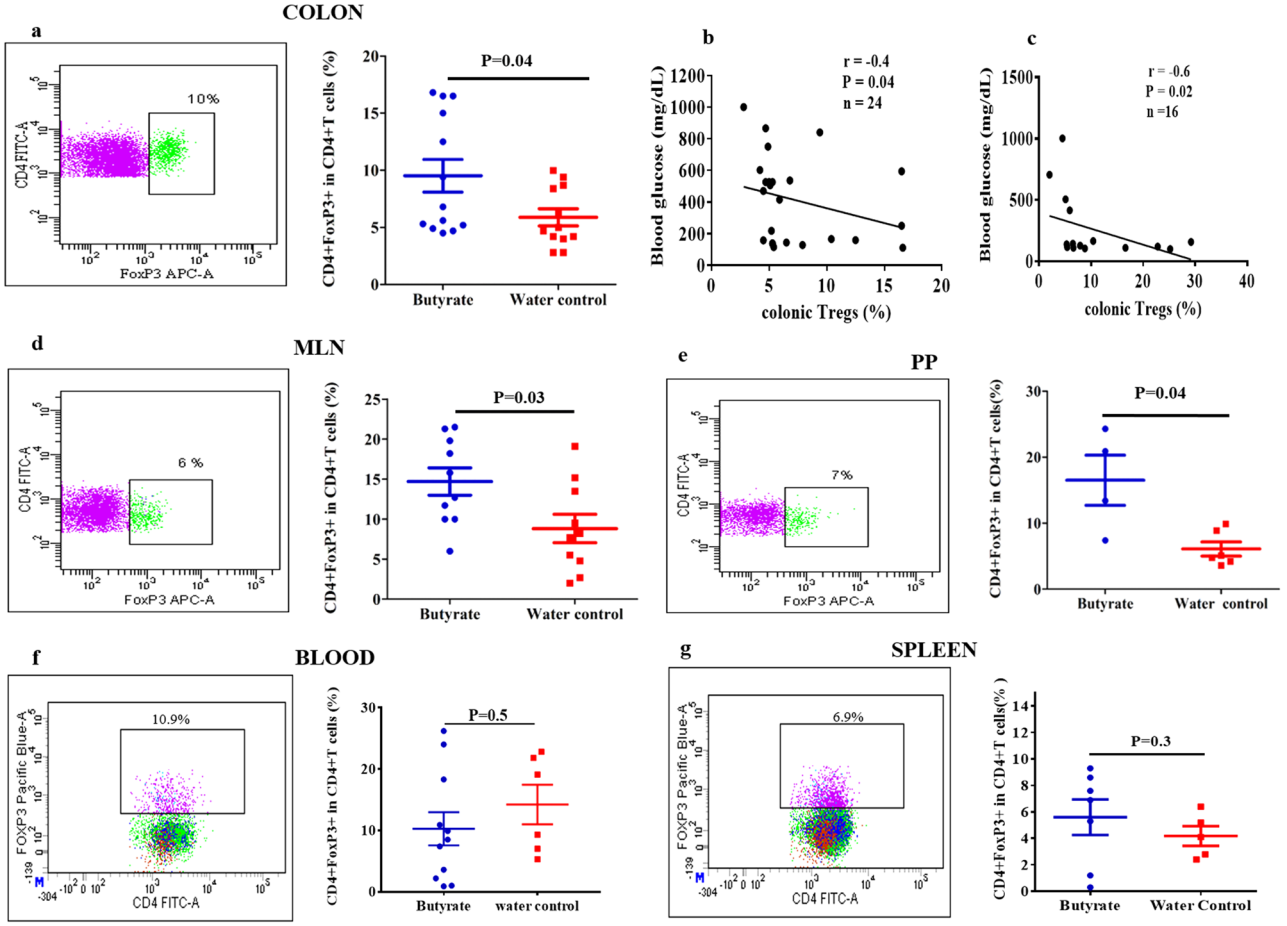
Butyrate treatment induces Tregs in the colon and GALT. Hyperglycemic NOD mice (age 15–25 weeks) were administered sterile drinking water with or without sodium butyrate (150 mM) for six weeks. The expression of CD4 + FoxP3 + Tregs from colonic lamina propria, Mesenteric lymph nodes (MLN), Peyer’s Patches (PP), Blood and Spleen were analyzed by flow cytometry. The gating strategy is shown in (Supplementary Fig S7). (**a**) Percent of CD4 + FoxP3 + T cells from colonic lamina propria in treatment and control groups (n = 12–13). Correlation between blood glucose levels (mg/dL) and percentages of cTregs in, (**b**) Hyperglycemic group (n = 24) post six weeks of treatment and, (**c**) Early-intervention group (n = 16) at 40 weeks of age were analyzed by Spearman’s rank correlation test. Percent CD4 + FoxP3 + T cells in (**d**) MLN (n = 10), (**e**) PP (n = 4–6), (**f**) Blood (n = 6–11) and (**g**) Spleen (n = 5–7). Lymphocytes were pre-gated on live CD3 + CD4 + T cells. Representative FACS plots are shown on the left and Treg frequencies (mean ± SEM) on the right. Statistical significance was determined by Mann–Whitney U test, with P < 0.05 considered as significant.

Few studies have demonstrated that modified diets yielding SCFA expanded the CD4 + FoxP3 + Treg cell population in NOD mice colon^16,17^. In line with these observations, we assessed if direct oral administration of butyrate was capable of generating cTregs. We observed increased frequencies of cTregs in the hyperglycemic NOD mice post butyrate treatment (P = 0.04) (Fig. 4a). Moreover, we investigated whether the induced Tregs in the colon can have an effect on the blood glucose levels. Hence, we performed a correlation analysis between the percentage of cTregs and blood glucose levels irrespective of the treatment and control groups. We observed a negative correlation between the percentages of cTregs and the blood glucose levels (r = − 0.4, P = 0.04) (Fig. 4b). A similar correlation was observed between cTregs and blood glucose levels in the early-intervention group as well (r = − 0.6, P = 0.02) (Fig. 4c). These results suggest that the induced Tregs in the colon could ameliorate diabetes in NOD mice.

Besides colon, MLN and PPs are the major sites of the gut mucosa for the induction of intestinal immune cells. T and B cells are activated in these mucosal sites and recent studies have shown that GALT plays an important role in islet-specific autoimmunity^13,18–20^. Therefore, in addition to cTregs, we also looked at the effect of butyrate treatment on the frequencies of induced CD4 + FoxP3 + Tregs in the, MLN and PP in hyperglycemic NOD mice. Tregs were isolated from the MLN and PP and results showed a significant increase in the frequencies of CD4 + FoxP3 + Tregs in MLN (P = 0.03) (Fig. 4d) and PPs (P = 0.04) (Fig. 4e) in comparison to the control group. These results suggest that butyrate treatment induces Tregs in all compartments of GALT. Since NOD mice received a continuous supply of butyrate through drinking water, we assumed that, apart from its effect on the GALT, butyrate could also have an effect on the Tregs in the spleen and blood. However, the treatment did not elevate the frequencies of CD4 + FoxP3 + Tregs in the treatment vs control groups in the blood (P = 0.5) (Fig. 4f) and spleen (P = 0.3) (Fig. 4g). Further analysis of the data in terms of absolute counts, again showed a significant increase in the CD4 + FoxP3 + Tregs in the colon, MLN and PP in the butyrate treated mice (Supplementary Fig S3).

### Butyrate protective effect is diminished upon depletion of Tregs

**Figure 5:**
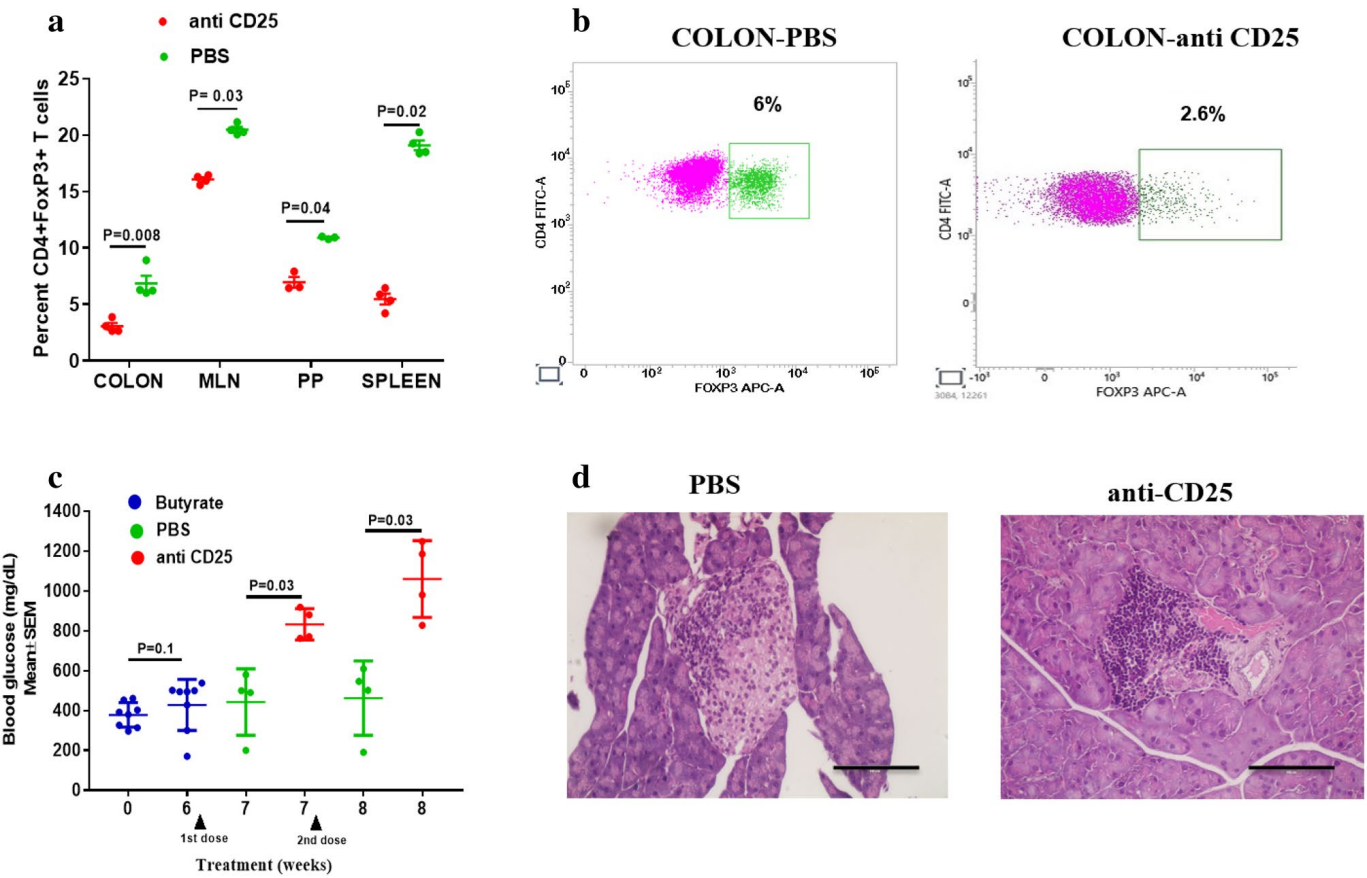
Abolishment of protective effect of butyrate upon Treg depletion. Hyperglycemic NOD female mice (n = 8) were treated with sodium butyrate (150 mM) in drinking water for 6 weeks and randomly divided into two groups. The treatment group (n = 4) received two intraperitoneal injections of anti-CD25 antibody (0.5 mg in PBS), while the control group (n = 4) received PBS alone, one week apart. (**a**) The frequency of Tregs (CD4 + FoxP3 + T cells) gated on live CD3 + CD4 + T cells in colon, MLN, PP and spleen were assessed by flow cytometry (n = 4 per group). (**b**) Representative flow cytometry plots show the frequency of CD4 + FoxP3 + T cells in colon of PBS treated and anti-CD25 treated NOD mice. (**c**) The graph shows the blood glucose levels of hyperglycemic NOD mice at day 0 and 6 weeks after butyrate treatment (n = 8). The graph also shows the blood glucose levels of mice treated with PBS (n = 4) and anti-CD25 (n = 4) at 7th (after the 1st dose of injections) and 8th week (after the 2nd dose of injections). (**d**) The pancreas were assessed for insulitis at 8th week post treatment in NOD mice. Representative photomicrographs of H&E stained pancreas sections of PBS administered (left) and anti-CD25 administered (right) groups at 40 × magnification; scale bars indicate 100 µm. Statistical significance was determined by Mann–Whitney U test, with P < 0.05 considered significant. Data are shown as mean ± SEM.

To understand whether the protective effects of butyrate i.e., reduction in hyperglycemia and insulitis were attained via Treg induction, we performed confirmatory experiments by depleting Tregs. Hyperglycemic NOD female mice were administered butyrate for 6 weeks. Further, the treatment group received two injections of anti-CD25 antibody at one week apart, while the control mice received an equal amount of sterile PBS. We observed a significant reduction in the percentages of CD4 + FoxP3 + Tregs in the colon (P = 0.008) (Fig. 5a,b) as well as other tissues including, MLN (P = 0.03), PP (P = 0.04), and spleen (P = 0.02) in the anti-CD25 administered group in comparison to the PBS treated group (Fig. 5a). Moreover, there was a significant reduction in the percentage of CD25 + FoxP3 + Tregs in all the GALT and spleen post anti-CD25 administration (Supplementary Fig S4). Further, we analyzed the effect of Treg depletion on the blood glucose levels in the hyperglycemic mice following butyrate treatment. Initially, there was no difference in the blood glucose levels on day 0 and after 6 weeks of butyrate treatment in NOD mice (P = 0.1) (Fig. 5c). However, after administration of anti-CD25, there was a significant increase in the blood glucose levels in the mice that received anti-CD25 (833 ± 39 mg/dL, mean ± SEM) in comparison to the control groups that received PBS injection (443 ± 83 mg/dL) (P = 0.03) (Fig. 5c). Similar results were observed following 2nd dose of anti-CD25 administration with mean blood glucose levels at 8th week being 462 ± 93 mg/dL in mice receiving PBS and 1060 ± 96 mg/dL in mice receiving anti-CD25 antibody (P = 0.03) (Fig. 5c). Moreover, we observed complete destruction (destructive insulitis) of pancreatic islets in mice that received anti-CD25, while mice that received PBS injection only showed partial destruction (Fig. 5d).

These results confirmed that the protective effect of butyrate was diminished upon depletion of Tregs.

### Butyrate treatment promotes the accumulation of gut homing receptor expressing Tregs in the PLN and Pancreas

**Figure 6:**
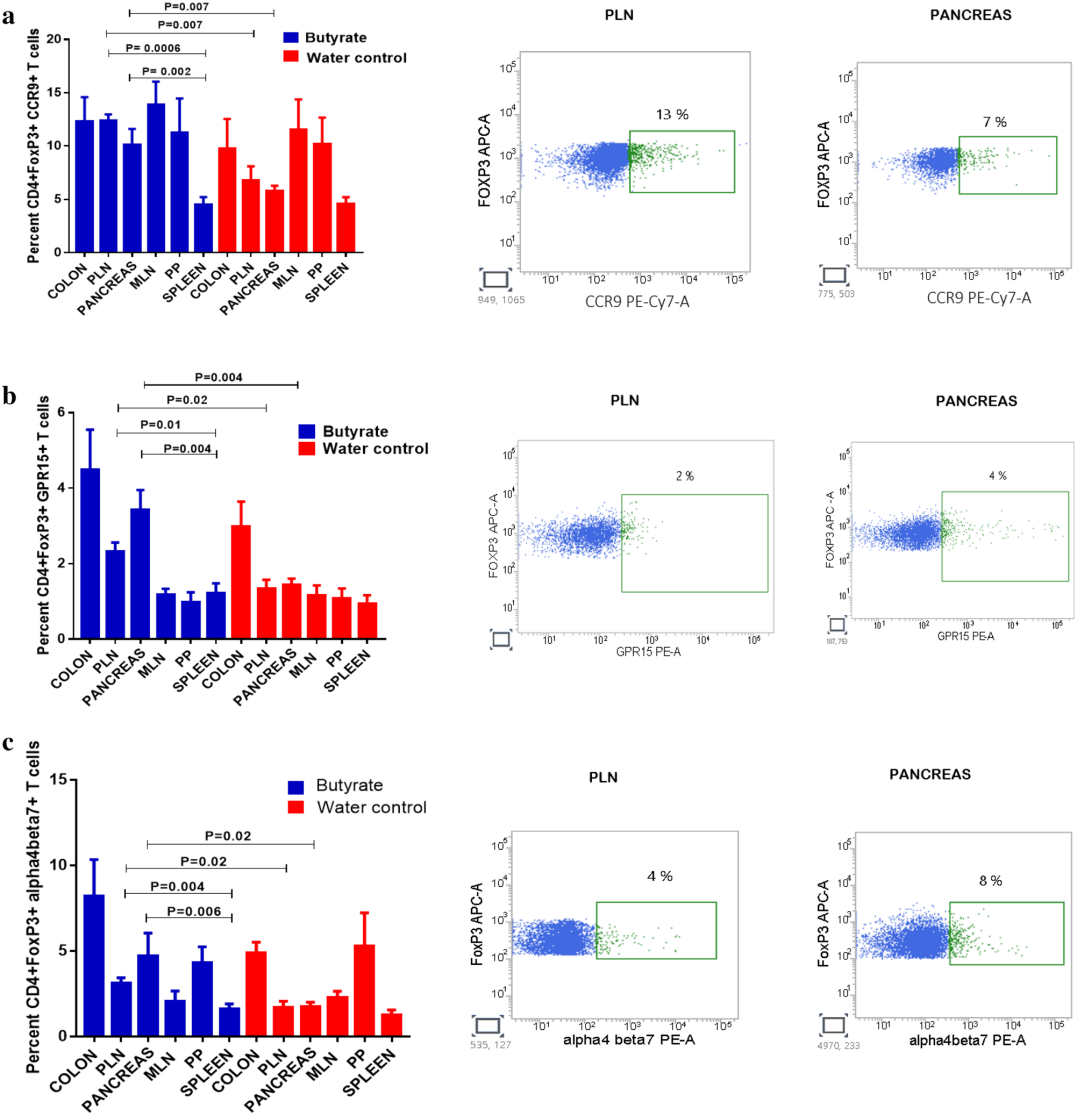
Butyrate treatment promotes the accumulation of gut homing receptor expressing Tregs in the PLN and Pancreas. Hyperglycemic NOD mice (age 15–25 weeks) were administered sterile drinking water with or without sodium butyrate (150 mM) for six weeks. Frequencies of Tregs expressing CCR9, GPR15 and alpha4beta7 were analyzed by flow cytometry. (**a**) CD4 + FoxP3 + CCR9 + Tregs in Colon (n = 5), PLN (n = 7), Pancreas (n = 7), MLN (n = 4), PP (n = 4), and Spleen (n = 7). (**b**) Percentage of CD4 + FoxP3 + GPR15 + Tregs in Colon (n = 4), PLN (n = 7), Pancreas (n = 7), MLN (n = 4), PP (n = 3–4) and Spleen (n = 7). (**c**) Percent of CD4 + FoxP3 + alpha4beta7 + Tregs in Colon (n = 4), PLN (n = 7), Pancreas (n = 7), MLN (n = 5–7), PP (n = 3) and Spleen (n = 7). Representative flow cytometry histograms showing CCR9 + Tregs, GPR15 + Tregs, α4β7 + Tregs from each tissue are shown on the right, pre-gated on live CD4 + FoxP3 + T cells. Results are displayed as mean ± SEM. Statistical significance was determined by Mann–Whitney U test, with P < 0.05 considered significant.

In order to gain mechanistic insights into the beneficial effects of butyrate induced Tregs in the GALT in diabetes remission, we first analyzed the expression of gut homing receptors, CCR9 (CD199), GPR15 and α4β7 in the Tregs in the GALT as well as the PLN, pancreas, and spleen in hyperglycemic NOD mice. In healthy humans and mice, T cell subsets that specifically express CCR9 migrate to the gut in response to the ligand CCL25. CCR9 + T helper cells are known to accumulate in the pancreas and other accessory organs of the digestive system during autoimmunity and other chronic inflammation^21^. Post-six weeks butyrate treatment of 15–25 weeks old hyperglycemic NOD mice, we observed a significant increase in the percentage of CCR9 expressing CD4 + FoxP3 + T cells in the PLN (P = 0.007) in comparison to untreated mice (Fig. 6a). Moreover, CCR9 expressing CD4 + FoxP3 + T cells were also increased in the pancreas of the treatment group (P = 0.007) (Fig. 6a). Most importantly, butyrate treatment also showed substantially higher levels of CCR9 + Tregs in the PLN (P = 0.0006) and pancreas (P = 0.002) in comparison to the spleen (Fig. 6a).

Another chemoattractant, GPR15 has also been identified as a gut homing receptor and SCFA stimulation has been shown to enhance the expression of GPR15 within the CD4 + Treg subset^22^. Hence, we examined the expression of GPR15 expressing Tregs in the GALT as well as the pancreas, PLN, and spleen. We observed that post butyrate treatment, CD4 + FoxP3 + GPR15 + Tregs were significantly increased in the PLN (P = 0.02) and pancreas (P = 0.004) (Fig. 6b). Further in comparison to the spleen as well, GPR15 expressing Tregs were significantly increased in PLN (P = 0.01) and pancreas (P = 0.004) (Fig. 6b).

The constitutive expression of MAdCAM-1, an adhesion molecule secreted by pancreatic islets allows homing of intestinal T cells that up-regulate α4β7 integrin on their surface following activation by gut-associated dendritic cells^11^. Post-six weeks treatment of 15–25 weeks old hyperglycemic NOD mice, a significant increase in the percentage of α4β7expressing CD4 + FoxP3 + T cells was observed in the PLN (P = 0.02) and the pancreas of the treatment group (P = 0.02) (Fig. 6c). Moreover, substantially higher frequencies of CD4 + FoxP3 + α4β7 expressing Tregs in the pancreas (P = 0.006) and PLN (P = 0.004) (Fig. 6c) in comparison to spleen further confirmed the accumulation of gut homing receptor expressing Tregs in the PLN and pancreas.

When we analyzed the data in terms of absolute counts, again, a significant increase in CCR9 + , GRP15 + , α4β7 expressing FoxP3 + Tregs was observed in the PLN and pancreas in butyrate treated mice in comparison to the spleen of butyrate treated mice (Supplementary Fig S5a,b,c). Further, to determine the expression of these gut homing receptors on per-cell basis we also analyzed the mean fluorescent intensity (MFI). The MFI of CCR9, GRP15, α4β7 on FoxP3 + Tregs was increased in the PLN and pancreas in the butyrate treated mice as well as in comparison to the spleen of butyrate treated mice (Supplementary Fig S5d,e,f).

Additionally, to evaluate whether Tregs expressing gut homing receptors can actually migrate towards the pancreas, we performed an in vitro transwell assay^23^. The colonic Tregs isolated from butyrate treated mice showed greater migration towards pancreatic homogenate as compared to the media control (MC) (P = 0.03) (Supplementary Fig S6a). These Tregs also showed greater chemotaxis towards CXCL12 which was used as a positive control (P = 0.03) (Supplementary Fig S6a). The fluorescence microscopy images of DAPI stained cells from the membrane of the transwell inserts also demonstrated greater chemotaxis of colonic Tregs towards CXCL12 as well as the pancreatic homogenate (Supplementary Fig S6b).

Overall, these results suggest that besides induction, butyrate treatment possibly improves the migratory potential of the colonic Tregs towards PLN and pancreas.

### Butyrate treatment induces expression of CXCL12 mediating T cell trafficking in the pancreas

**Figure 7:**
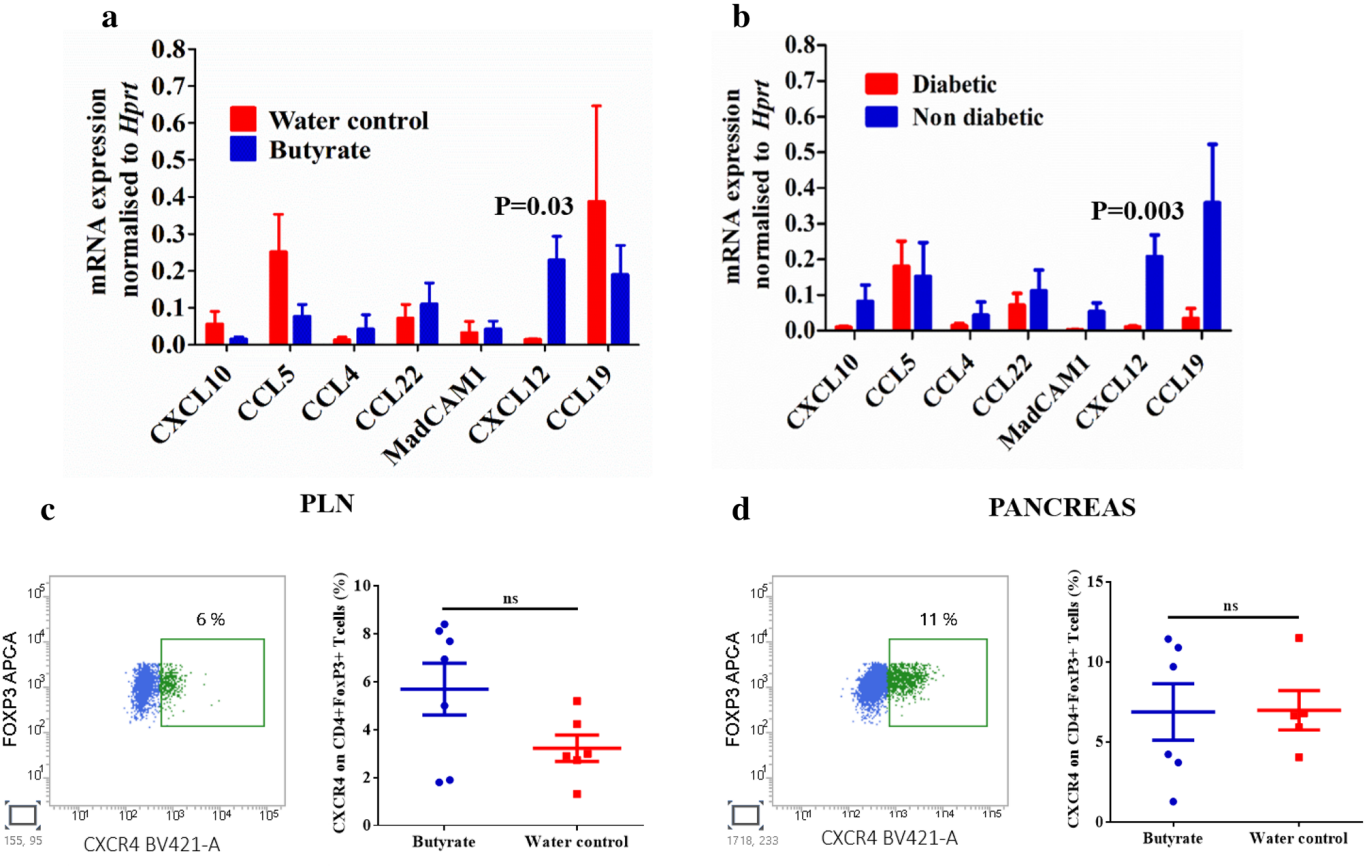
Expression of chemokines and chemokine receptors involved in Treg trafficking. Hyperglycemic NOD mice (age 15–25 weeks) were administered sterile drinking water with or without sodium butyrate (150 mM) for six weeks. (**a**) The expression of chemokines CXCL10, CCL5, CCL4, CCL22, CXCL12, MAdCAM1, CXCL19 was analyzed in the pancreas in the treatment and control groups by quantitative RT-PCR (n = 6–13 mice per group). (**b**) The expression of chemokines in the diabetic versus the non-diabetic mice (n = 6–13 mice per group). Error bars indicate mean ± SEM. The data were expressed as 2^−ΔΔCT^ and each gene was normalized with internal control gene HPRT. The frequency of chemokine receptor CXCR4 expressed on CD4 + FoxP3 + Tregs in (**c**) PLN (n = 6–7 mice per group) and (**d**) pancreas (n = 5–6 mice per group) was assessed by flow cytometry. Representative flow cytometry histograms showing CXCR4 + FoxP3 + T cells from pancreas and PLN are shown on the left, pre-gated on live CD4 + FoxP3 + T cells and cell frequencies (mean ± SEM) on the right. Statistical significance was determined by Mann–Whitney U test, with P < 0.05 considered significant.

The Treg cells, like any other cell type, migrate to the tissue of their destination due to the presence of chemokines in the local tissue and the expression of corresponding receptors on their surface. Therefore, we explored the chemokine expression in the pancreas, which are involved in the trafficking of Tregs. The pancreas tissue was collected from NOD mice after six weeks of treatment with butyrate along with their respective control groups. Quantification of gene expression of CXCL10, CCL5, CCL4, CCL22, CCL19, CXCL12 and MAdCAM1 was performed by RT-PCR. Of all the chemokines analyzed, the expression levels of CXCL12 was significantly higher (P = 0.03) in the butyrate-treated group (Fig. 7a). Whereas, we could not find any significant change in the expression of CXCL10, CCL5, CCL4, CCL22, CCL19, and MAdCAM1 between the treated and control NOD mice (Fig. 7a). Additionally, when we included non-diabetic control mice and compared the expression of various chemokines in the pancreas with the diabetic NOD mice, we observed a relatively higher expression of CXCL12 in the non-diabetic NOD mice (P = 0.003) (Fig. 7b). Further, we examined the distribution of CXCR4 expressing CD4 + FoxP3 + Tregs in the PLN and pancreas by flowcytometric analysis and observed that CXCR4 was indeed expressed in the Tregs in the PLN and pancreas of NOD mice (Fig. 7c,d). On analysis of MFI data, we also observed augmented expression of CXCR4 in the Tregs of pancreas (P = 0.4) and PLN (P = 0.1) in the butyrate treated mice, though it was not significantly higher than the water control group (Fig S5g). Nevertheless, these results highlight the importance of CXCR4/CXCL12 pathway in protection against T1D.

### Butyrate induced Tregs can migrate towards the pancreas and PLN and delay the onset of diabetes

**Figure 8:**
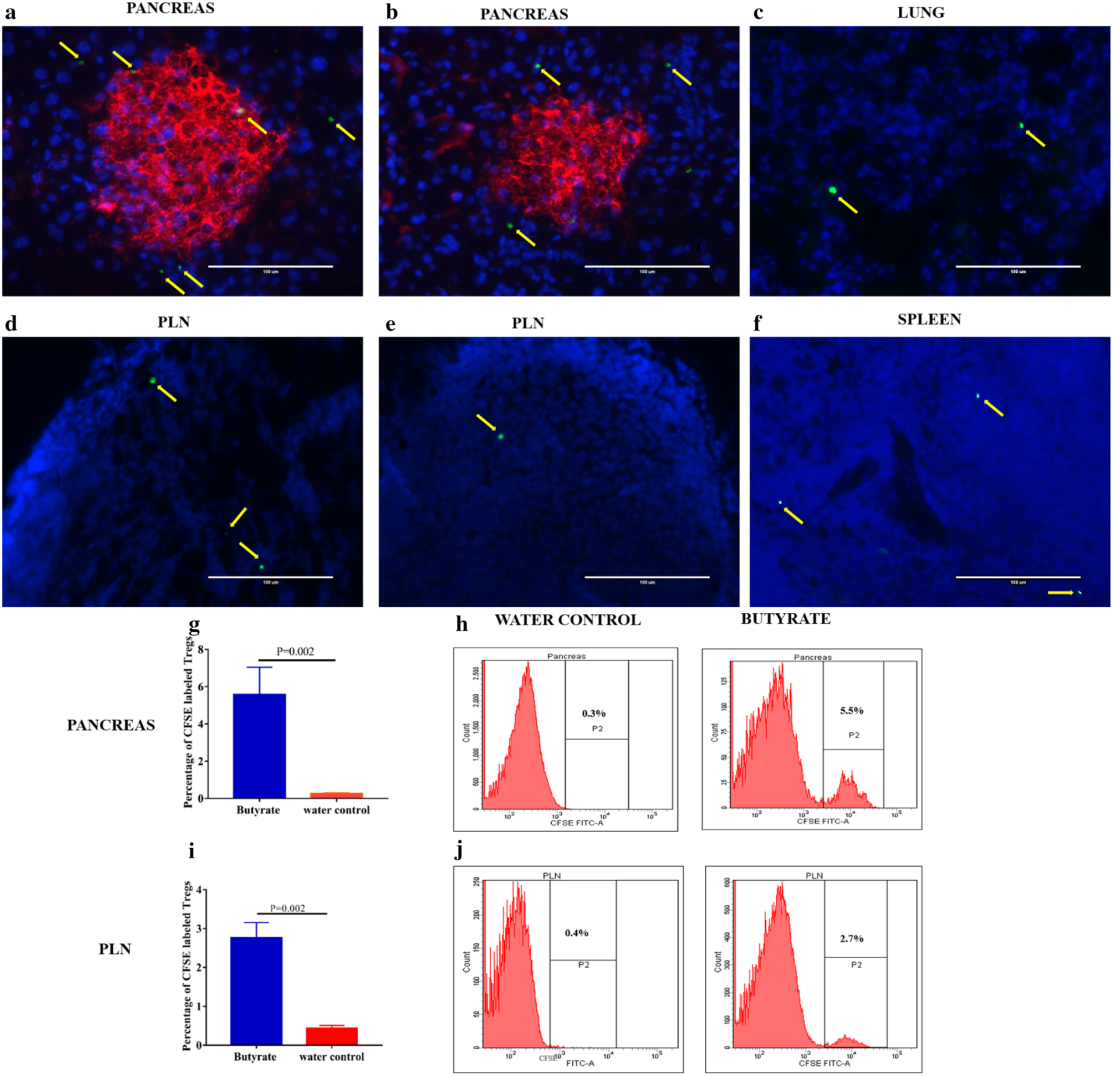
Butyrate treatment promotes the migration of induced Tregs from the GALT to the pancreas and pancreatic lymph nodes. Hyperglycemic NOD mice (age 15–25 weeks) were given sterile drinking water with or without sodium butyrate (150 mM) for 6 weeks. CD4 + CD25 + Tregs were isolated from the MLN, PPs, and colon, CFSE labeled and intravenously injected into 20 weeks old recent-onset diabetic (recipient) NOD mice. Photomicrographs of sections of (**a**) Pancreas and, (**d**) PLN from recipient NOD mice show the migration of CFSE labeled CD4 + CD25 + Tregs from butyrate treated NOD mice after 5 days of adoptive transfer. Photomicrographs of sections of (**b**) Pancreas and, (**e**) PLN from recipient NOD mice show the migration of CFSE labeled CD4 + CD25 + Tregs from control NOD mice after 5 days of adoptive transfer. Photomicrographs of sections of (**c**) Lung and (**f**) spleen tissue show the migration of CFSE labeled CD4 + CD25 + Tregs from the butyrate-treated mice to the recipient NOD mice. The arrows indicate CFSE labeled CD4 + CD25 + T cells (green). The pancreatic and PLN sections were stained for DNA with DAPI (blue). The islets in the pancreatic sections are represented by insulin staining (Cy-3, red). The slides were viewed on the fluorescence microscope and images were visualized using Pearlscope software at 40 × magnification; scale bars indicate 100 µm. The frequency of migrated CFSE labeled CD4 + CD25 + Tregs (among total CD4 + T cells isolated from recipient pancreas or, PLN) from butyrate treated and control mice into the (**g**) Pancreas and, (**i**) PLN of recipient hyperglycemic NOD mice was quantitatively assessed by flow cytometry. The representative flow cytometry histograms of (**h**) Pancreas and, (**j**) PLN are shown on the right. Results are displayed as mean ± SEM (n = 6 mice per group). Statistical significance was determined by Mann–Whitney U test, with P < 0.05 considered significant.

Next, to evaluate whether induced Tregs in the GALT have the potential to migrate to the pancreas and PLN, we performed adoptive transfer experiments. CFSE labeled CD4 + CD25 + Tregs isolated from the MLN, PPs, and colon of butyrate-treated hyperglycemic NOD mice were intravenously injected into 20 weeks old recent-onset diabetic NOD mice (recipient mice). After 5 days of the adoptive transfer, the immunofluorescence images of the pancreas and the PLN sections revealed the migration of Tregs obtained from butyrate-treated mice towards the pancreas and PLN of the recipient mice (Fig. 8a,d). The CFSE labeled Tregs transferred from the control groups showed less migration towards the pancreas and PLN (Fig. 8b,e) in the recipient diabetic NOD mice. Further, the specificity of Treg migration was confirmed by assessing the presence of CFSE labeled CD4 + CD25 + Tregs in the lung (Fig. 8c) and splenic (Fig. 8f) tissue in recipient mice that received Tregs from butyrate treated mice.

In addition to immunofluorescence, we determined the frequency of CFSE labeled Tregs in the pancreas and PLN of recipient NOD mice by flow cytometry. The Tregs adoptively transferred from butyrate-treated NOD mice showed greater migration towards pancreas (5.5 ± 1.5%, mean ± SEM) (P = 0.002) (Fig. 8g,h) and PLN (2.8 ± 0.4%, mean ± SEM) (P = 0.002) (Fig. 8i,j) in hyperglycemic recipient mice as compared to the CFSE labeled Tregs transferred from the control groups. Since the Tregs were intravenously injected into NOD mice, we also examined the migration of Tregs isolated from the spleen of butyrate-treated mice (as a control) towards the pancreas and PLN. We observed a significantly higher migration of Tregs isolated from the GALT to the pancreas (P = 0.01) and PLN (P = 0.01) in comparison to the Tregs isolated from the spleen (Supplementary Fig S7a-d). These results confirmed that butyrate induced Tregs in the GALT, preferentially migrate towards the pancreas and PLN of diabetic NOD mice.

Further, we wanted to assess whether these butyrate induced Tregs have the potential to delay the onset of diabetes. Hence, we adoptively transferred CD4 + CD25 + Tregs isolated from the GALT of butyrate treated and water control NOD mice to 6–8 weeks old female NOD mice (n = 15 per group). We observed a significant delay in the onset of diabetes in those mice that received butyrate induced Tregs (mean ± SEM, 34.3 ± 2.3 weeks) in comparison to the mice that received Tregs from the control mice (19.8 ± 1.4 weeks) (p = 0.006) (Fig S7e). However, we could not find a significant difference in the frequency of diabetes development in recipient mice that received Tregs from the butyrate treated and control mice. Four out of 15 (27%) mice that received butyrate induced Tregs and 7 out of 15 (47%) of mice that received Tregs from the water control group developed diabetes (p = 0.3) until the end of 40 weeks (Fig S7f). Overall, these results indeed suggest that butyrate induced Tregs are capable of delaying the onset of diabetes.

## Discussion

The present study highlights the importance of butyrate in delaying the onset and progression of diabetes in NOD mice via induced Tregs in the GALT. It has been demonstrated that the gut microbiota conditions the immune environment in the pancreas through SCFA thereby controlling autoimmune diabetes. Alterations in the SCFA milieu has been observed in the intestine of diabetic subjects, with the individuals who do not develop diabetes having higher levels of butyrate^24^. However, the mechanisms by which SCFA like butyrate confer tolerance in the pancreas and PLN are not completely understood. Butyrate yielding diets have been shown to significantly augment the frequencies of colonic Tregs^17^. Hence, the use of postbiotics such as butyrate offers a therapeutic possibility for the treatment of T1D.

In order to replicate trials in humans with established diabetes, such as the Diabetes Prevention Trial 1 or the oral insulin trial^25,26^, late prevention strategies or reversal studies have been carried out, but with limited success. In this context, we investigated the efficacy of sodium butyrate treatment post-hyperglycemia (reversal study) with the rationale that butyrate might halt further damage to the islets. We observed nearly 33% of diabetes remission in the butyrate-treated group. At the same time, the blood glucose levels in the rest of the treated mice did not progress to the levels seen in the untreated control group. We also observed better survival of the treatment group, indicating a beneficial effect of butyrate treatment. We further investigated its effects on the suppression of insulitis. In NOD mice by around 10–12 weeks of age insulitis becomes well established. There is variability in the extent of insulitis in different islets with heavily infiltrated islets co-existing with functionally intact islets^27^. In our study butyrate treatment prevented the progression of insulitis and even ameliorated insulitis in nearly 50% of the islets. In comparison to NOD mice, the autoimmune process in humans is delayed and the clinical onset of the disease occurs once 80% or more beta cells are destroyed^28^. This provides a window of opportunity for providing therapeutic intervention in pre-diabetic individuals. Since the tussle between autoreactive T cells and the Tregs actively occurs during the pre-clinical stage before the onset of insulitis, we also initiated butyrate treatment at 4 weeks of age in NOD mice. Recently, a study by de Groot et al. (2020), showed that oral supplementation of sodium butyrate in long-standing T1D did not affect the adaptive or innate immune systems^29^. On the positive side, this study opens up the possibility of exploring the beneficial effects of butyrate by initiating intervention as early as possible. Interestingly, our data from the early-intervention in NOD mice show delay in the onset of diabetes along with the reduction in insulitis, indicating that such interventions could be more beneficial when initiated as early as possible, such as in high-risk individuals or new-onset T1D. Additional modulatory approaches can be added to augment and prolong the beneficial effects of butyrate and counter the growing effector T cell responses during the disease progression. Also, early interventions give more time for the accumulation of beneficial effects, unlike short-term therapeutic interventions as performed by Groot et al.

Next, the GC–MS analysis in our study clearly showed that the observed effects in butyrate treated mice could be attributed to oral administration of butyrate. However, the contribution of endogenous butyrate and other SCFA produced by gut-resident microbiota in Treg induction cannot be ruled out. It is also known that SCFA can potentiate insulin secretion by acting on GPR43 present on the beta cells^30^. In the present study, it is possible that butyrate could have promoted insulin secretion via acting on this receptor in the early-intervention group. However, insulin staining and insulitis scores of the islets in the hyperglycemic treated mice suggested increased numbers of functional beta cells that were prevented from destruction following butyrate treatment. Further, the improvement in the C-peptide levels indicates that the residual beta cell mass is capable of insulin secretion that could lower the blood glucose levels to an appreciable extent.

GALT harbors the largest frequency of peripheral Tregs (pTregs). The colonic lamina propria has around 25–30% of CD4 + T cells, which are FoxP3 + pTregs^31^. The present study demonstrated the ability of butyrate to induce cTregs, was associated with a decline in blood glucose levels, suggesting that the suppressive peripheral tolerance could prevent the diabetogenic T cells from entering the pancreatic islets. Moreover, the transient depletion of these butyrate induced Tregs by anti-CD25 administration^32^ abolished their protective effect as indicated by uncontrolled hyperglycemia in these NOD mice. MLN are gut-draining lymph nodes and the DCs migrate towards the MLN from the gut to promote pTreg generation^33,34^. In this study butyrate treatment demonstrated a strong abundance of Tregs in the MLN and PP which suggests that GALT plays an important role in islet-specific autoimmunity. The proximity of the gut with pancreas or PLNs has been evidenced by the induction of diabetes by the T cells transferred from MLN of pre-diabetic NOD mice into NOD SCID recipients^35^. Consequently, we postulated that Tregs from the gut might migrate to the PLN and pancreas via blood circulation in response to butyrate treatment. Previous studies have reported that the T cells infiltrating the pancreatic islets express the gut-homing receptor α4β7 integrin. Also, the expression of MadCAM-1 the specific ligand for this integrin in the pancreas confirms its involvement in lymphocyte trafficking^13,18,36^. The gut homing receptor CCR9 expressing Th cells derived from the PP and MLN has been shown to migrate to inflamed regions of the pancreas in NOD mice. Moreover, its ligand, CCL25 has been shown to be expressed in the pancreas for pre-diabetic NOD mice^21,37^. GPR15 is another orphan G protein coupled receptor specifically controlling the migration of FoxP3 + Tregs into colonic lamina propria and is known to maintain mucosal immune tolerance^15^. Therefore, we included all of these markers to detect the trend of migration of Tregs from the colon, MLN and PP. Post-treatment the percentage of α4β7 + , CCR9 + , GPR15 + cells among the CD4 + Foxp3 + in PLN and pancreas increased, indicating that these Tregs primarily originated from the gut and moved to these tissues in response to inflammatory stimuli.

The Treg accumulation pattern in the pancreas or PLN is governed by the expression of various adhesion molecules, chemokine ligands, and their respective integrins or chemokine receptors on the Tregs^38^. Studies have shown decreased expression of CXCL12 in the PLN correlated with the lack of Tregs in diabetic NOD mice^8^. In our study, increased expression of CXCL12 in the pancreas in the treatment group along with the expression of their receptor CXCR4 in the PLN and pancreas suggests the importance of CXCL12/CXCR4 pathway as a tool to examine the Treg dependent immunity and its ability to prevent autoimmune diabetes. This observation also opens up another question, as to how butyrate could increase the expression of these chemokine receptors on cTregs and their ligands in the pancreatic tissue. Finally, the adoptive transfer experiments revealed the presence of these induced Tregs in the pancreas and PLN further confirming the migratory potential of butyrate induced Tregs from the GALT to the site of inflammation. A recent approach of co-transfer of CD4 + CD49b + LAG3 + Tr1 cells along with diabetic splenocytes showed a significant decrease in diabetes in NOD mice^39^. Such studies add to the evidence that a combined therapy that increases Tregs in the pancreas either directly or indirectly can induce tolerance in human T1D.

We used only sodium butyrate and did not combine it with any other therapeutic agent. Since the onset of autoimmune diabetes depends on various islet-specific factors and effector T cells that cause beta cell damage appear later, therefore, butyrate treatment alone may not have much impact on the initial islet priming events. Nevertheless, it is still possible that the administration of sodium butyrate along with a beta cell regenerative or beta cell-specific immunosuppressive agent and longer duration of treatment might exert better protection against the disease. Further, it remains to be explored whether butyrate modulates other cell types such as effector CD8 + T cells, NK cells or Th17 cells. To conclude, our study demonstrates that **s**odium butyrate plays an immunomodulatory role via induction of Tregs in the GALT that are capable of migrating towards the pancreas and PLN, thereby leading to diabetes remission. Such approaches provide new options in the treatment of T1D that are safe and tolerogenic.

## Materials and methods

### Mice

Female NOD mice (strain NOD/MrkTac**)** were purchased from Taconic Laboratories, New York, USA and housed in individually ventilated cages (IVC) in the Small Animal Facility at Post Graduate Institute of Medical Education and Research (PGIMER) and iCARE, Institute of Microbial Technology (IMTech), Chandigarh, India. All animals were maintained at 12 h light–dark cycle and temperature between 20–25 °C. The animals were fed with a maintenance diet for mice (ATNT Laboratories, Mumbai, India) and sterile water ad libitum throughout the experiments. All experiments were performed in accordance with the protocol approval from the Institutional Animal Ethics Committee (IAEC), PGIMER. The incidence of diabetes was monitored by taking weekly blood glucose measurements from the tail vein using Glucometer (Abbott FreeStyle Optium, United States). Serum samples were stored at − 80 °C for further analysis, including the determination of C-peptide and insulin by ELISA (Krishgen Biosystems, Mumbai, India). Mice were considered diabetic when blood glucose levels were more than 250 mg/dL for two consecutive days.

### Sodium butyrate treatment

The mice were divided into two groups, the treatment and control groups. In the treatment group, hyperglycemic NOD female (age 15–25 weeks) were treated with sodium butyrate (Himedia, Mumbai, India) (150 mM)^4^ in drinking water, while the control hyperglycemic NOD mice received an equal volume of sterile water (n = 10–15). The mice were sacrificed at 25 weeks post hyperglycemia or at the maximum point of survival. In addition, treatment was initiated in an early-intervention group where NOD female mice (age 4 weeks) received sodium butyrate in drinking water (treatment group) or sterile drinking water (control group) (n = 17–20) until 40 weeks of age. The mice were sacrificed at the onset of diabetes or, at 40 weeks of age.

### Histology

The pancreas was isolated and fixed in 10% phosphate-buffered formalin (nine parts of phosphate buffer saline in one part of 40% formaldehyde) and embedded in paraffin. Sections were cut at the 5 µm thickness, with a minimum of three non-consecutive sections at 70 µm apart, deparaffinized and stained with hematoxylin and eosin (H–E; Merck, Whitehouse Station, NJ, USA). The infiltration of mononuclear cells in the islets was observed under the light microscope (Leica DFC 295, Wetzlar, Germany) using Leica Application Suite version 3.7.0 at an original magnification of 20 × or 40 × and graded in a blinded fashion. The degree of insulitis was scored according to the following criteria: Score 0, no insulitis; Score 1, peri-insulitis; Score 2, mild insulitis (less than 25% infiltration); Score 2, severe insulitis (25% to 75% infiltration); Score 3, destructive insulitis (more than 75% infiltration) (Supplementary Fig S1). The insulitis index was calculated according to the formula; Insulitis index = (0 × n_0_) + (1 × n_1_) + (2 × n_2_) + (3 × n_3_) + (4 × n4) / 4 (n_0_ + n_1_ + n_2_ + n_3_ + n_4_), where n_0_–n_4_ denotes the number of islets of scores from 0 to 4^40^.

The histomorphometric analysis of insulin was performed by indirect immunohistochemistry. The pancreatic sections were deparaffinized and rehydrated; antigen retrieval was done in sodium citrate buffer (pH 6). Following blocking with 3% H_2_O_2_ for 45 min, the pancreatic sections were incubated with blocking buffer (1% BSA in PBS) for 2 h. The sections were incubated with mouse insulin antibody (1:1000 in blocking buffer) (Cell Signaling Technology, Massachusetts, USA) at 4 °C overnight. Following washing, the sections were stained with horseradish peroxidase-conjugated secondary antibody (Goat Anti- Mouse IgG HRP conjugate, 1:500 in blocking buffer, Biorad, California, USA), for 1 h. Beta cell (cells stained for insulin) area occupied by the islets were calculated by the percentage ratio of insulin-cell stained area to the total area of the same islet (ImageJ software 1.48v, Image Processing, and Analysis in Java, NIH, USA). The images were acquired at 20 × and 40 × magnification.

### RNA isolation and cDNA synthesis

TRIzol-chloroform-isopropanol method was used to isolate RNA from mice pancreas, followed by purification through a column using the RNeasy Mini kit (Qiagen GmbH, Hilden, Germany) according to the manufacturers' instructions. One µg of the extracted RNA was reverse transcribed using REVERT-AID First Strand cDNA Synthesis Kit (Thermo Scientific, Massachusetts, USA) according to the manufacturer’s protocol.

The quantitative real-time PCR was performed on real-time PCR (Step One Plus, Applied Biosystems, California, USA) using TaqMan Universal PCR Master Mix (Applied Biosystems). The primers and probes for the quantitative detection of various chemokines such as CXCL10, CCL5, CCL4, CCL22, CXCL12, MAdCAM1, CXCL19 and Hypoxanthine–guanine phosphoribosyl transferase (HPRT) (internal control gene) were synthesized (Applied Biosystems). The assay IDs for various chemokine genes are shown in (Supplementary Table S2). The results were analyzed using the StepOne Software v2.3 (Applied Biosystems). The data were expressed as 2^−ΔΔCT^to determine the relative expression of the chemokines, where ΔC_T_ for each sample was determined by subtracting the gene threshold (C_T_) value of HPRT from the C_T_ for genes. The ΔΔC_T_ was calculated by subtracting the ΔC_T_ for each treated sample from the ΔC_T_ of the untreated control sample.

### Colonic lamina propria lymphocyte isolation

Colon was isolated from NOD mice and opened longitudinally, washed with 1X PBS to remove fecal contents and cut into 0.5 cm pieces. The epithelial cells, mucus, and fat tissue, from the colon, were removed by incubating in 1X HBSS (Sigma-Aldrich, St. Louis, MO, USA) containing 5 mM EDTA (Sigma-Aldrich) and 1mMDTT(dithiothreitol) (BioRad) for 30 min at 37 °C. Colon was then minced and digested using digestion solution containing 1 mg/mL collagenase VIII (Sigma-Aldrich), 0.5 mg/mL DNase (MP Biomedicals, Santa Ana, CA, USA), and 2% FBS (Thermo Scientific) in 10 mL of RPMI (Sigma-Aldrich) for 30 min at 37 °C at 200 rpm. The crude cell suspension was resuspended in 10 mL of 40% Percoll fraction and overlaid in 5 mL of 80% Percoll fraction (MP Biomedicals) and centrifuged at 1000 *g* for 20 min at room temperature with brake turned off^41^. Lamina Propria mononuclear cells (LPMCs) were collected from the white ring at the interphase of the Percoll solution.

### Flow cytometry analysis

Single-cell suspension obtained from various tissues were stained with fixable viability dye (FVD) (Zombie NIR Fixable Viability Kit) (Biolegend, San Diego, California, United States) in staining solution (PBS containing 2% FBS and 1 mM EDTA) for 5 min. For Tregs staining, the cells were first stained with surface antibodies for 20 min at room temperature. The intracellular staining for FoxP3 was performed by fixing and permeabilizing the cells using TRUE-NUCLEAR transcription factor buffer set (Biolegend) according to manufactures protocol and then staining for FoxP3. The following anti-mouse antibodies were used, CD3-PerCP (145-2C11, Biolegend), CD4-FITC (GK1.5, BD Biosciences), CD4-APC-Cy7 (GK1.5, BD Biosciences), CD25-PE (PC61, BD Biosciences), FoxP3-APC (JFK-16s, eBioscience), FoxP3-BV421 (MF23, BD Biosciences), LPAM-1 (α4β7)-PE (DATK32, BD Biosciences), CD184 (CXCR4)-BV421 (2B11, BD Biosciences), CD199 (CCR9)-PE/Cy7 (CW-1.2, Biolegend), GPR15/BOB-PE (S150421, Biolegend). Fluorescent minus one (FMO) tubes were used as controls for positive gating in the case of FoxP3, α4β7, CCR9, GPR15 and CXCR4. Flow cytometry was performed using BD FACS Canto II flow cytometer and analyzed with BD FACS DIVA software, version 6.2.1 (BD Biosciences). The gating strategy of induced Tregs is shown in (Supplementary Fig S8).

### Transwell migration assay

Migration assay was performed using 5-µm polycarbonate filter inserts in a 24 well plate (BD Biosciences)^23^. The NOD mice pancreas was homogenized using a laboratory homogenizer in a 5 ml of RPMI 1640 medium. Samples were centrifuged at 18,000 *g* to eliminate cell debris. Briefly, 650 µL of pancreatic extract in complete RPMI 1640 medium was added to the lower chamber of the 24 well plate. CD4 + CD25 + Tregs were isolated from the colon of butyrate treated NOD mice using EASYSEP mouse CD4 + CD25 + regulatory T cell isolation kit II (Stem Cell Technologies, Vancouver, British Columbia). Around 1.5 × 10^5^ CD4 + CD25 + T cells in 200 µL of complete RPMI medium were added to the upper chamber of the transwell insert. 650 µL of complete RPMI containing recombinant purified CXCL12 (400 ng/mL) (Peprotech, USA) was added to the lower chamber as a positive control. Complete RPMI with the absence of any chemokine or pancreatic extract was used as a negative control (media control). Transwell assay was performed in 37 °C in a humidified 5% CO_2_ incubator for 24 h in triplicate wells. After incubation the inserts were removed and the migrated cells were recovered from the lower compartment and stained with anti-CD4-FITC and anti- CD25-PE mAb. The frequencies of migrated cells were assessed by flowcytometry. Consequently, the cells that were migrating through the membrane of the inserts were stained with DAPI and visualized using a Fluorescent microscope. For fluorescent microscopy one of the inserts from triplicate was taken after 2 hours of incubation to show the migration in process. The images were visualized using Pearlscope software at 40 × magnification.

### Treg depletion assay

Hyperglycemic NOD mice (n = 8) were treated with sodium butyrate (150 mM) for six weeks. Further, they were randomly divided into two groups. Treatment group received two intraperitoneal injections (0.5 mg in 1XPBS) of anti-CD25 antibody (clone PC61, Bioxcell, NH, USA) one week apart (n = 4)^32^. Control mice received the same dose and injection schedule of 1X PBS (n = 4). Blood glucose was estimated before and after the butyrate treatment as well as post anti-CD25 or PBS administration.

### Adoptive transfer experiments for Treg migration

15–25 weeks old hyperglycemic NOD mice after treatment with sodium butyrate or sterile water for 6 weeks were sacrificed and the Tregs were isolated from the MLN, PPs, and colon using EASYSEP mouse CD4 + CD25 + regulatory T cell isolation kit II (Stem Cell Technologies, Vancouver, British Columbia). CFSE (Carboxyfluorescein succinimidyl ester) (Biolegend) labeled CD4 + CD25 + T cells (1 × 10^6^cells) were adoptively transferred into diabetic NOD mice via tail vein injection. After 5 days of the adoptive transfer, the pancreas, PLN and lung tissue were isolated from recipient mice and analyzed for the migration of CFSE labeled cells. The tissues were cryosectioned at 5 µm thickness. The Nuclear staining was achieved by Nuclear dye 4′,6-diamidino-2-phenylindole (DAPI) (ProLong Gold Antifade Mountant with DAPI, Thermoscientific). For the pancreatic sections, primary antibody used was mouse insulin antibody (1:100; Cell Signaling Technology), the secondary antibody used was Cy-3 Goat anti mouse (1:200) (Abcam, UK). The slides were viewed on a Fluorescence microscope (Olympus BX63F, Shinjuku, Tokyo, Japan) and images were visualized using GenASIs FISH View software. The images were taken at 40 × magnification. The CFSE labeled cells from pancreas and PLN of recipient mice were also analyzed by flow cytometry.

### Adoptive Tregs transfer for diabetes prevention

CD4 + CD25 + regulatory T cells were isolated from the GALT of butyrate treated or water control NOD mice by EASYSEP mouse CD4 + CD25 + regulatory T cell isolation kit II. A total of 5 × 10^6^ cells (pooled from 2 mice) were intravenously injected into 6–8 weeks old female NOD mice (n = 15 per group) through the tail vein. A second intravenous injection of Tregs was given on day 6 after the initial intravenous injection. Another booster of intravenous injection of Tregs was given on day 12 post transfer^42^. Recipient mice were followed for diabetes onset until 40 weeks of age.

### Statistical analysis

The data were analyzed using Graph Pad Prism software version 7.0 (GraphPad Software, La Jolla, CA, USA). D’Agostino and Pearson Omnibus normality test was done to check the normal distribution of data. Two independent groups were compared using the two-tailed unpaired-test (when the data is normally distributed) or nonparametric Mann–Whitney t-test. The correlation between two independent variables was calculated using Pearson’s and Spearman’s rank correlation coefficient. The results were represented as mean ± standard error of mean (SEM). The survival of mice was graphed using Kaplan–Meier survival plots and was analyzed using the Mantel-Cox log-rank test. A p-value of less than 0.05 was considered significant for all analyses.

### Conference presentation

A portion of this work has been presented in the 16th International Congress of the Immunology of Diabetes Society (IDS 2018), 25–29 Oct 2018, London, UK.

## Supplementary information

Supplementary Information.
