# Supplementary material for: Butyrate induced Tregs are capable of migration from the GALT to the pancreas to restore immunological tolerance during type-1 diabetes
Source: Sci Rep. 2020 Nov 5;10:19120. doi: 10.1038/s41598-020-76109-y (PMC7644709; doi:10.1038/s41598-020-76109-y)
Supplement: Supplementary file 1 — Supplementary Information. [file 41598_2020_76109_MOESM1_ESM.docx]

**Butyrate induced Tregs are capable of migration from the GALT to the pancreas to restore immunological tolerance during type-1 diabetes**

Neenu Jacob^1^, Shivani Jaiswal^2^, Deep Maheshwari^2^, Nayudu Nallabelli^2^, Neeraj Khatri^3^, Alka Bhatia^4^, Amanjit Bal^5^, Vivek Malik^6^, Savita Verma^1^, Rakesh Kumar^1^ and Naresh Sachdeva^2*^

^1^Department of Pediatrics, Post Graduate Institute of Medical Education and Research (PGIMER), Chandigarh, India

^2^Department of Endocrinology, Post Graduate Institute of Medical Education and Research (PGIMER), Chandigarh, India

^3^iCARE, Institute of Microbial Technology (IMTech), Chandigarh, India

^4^Department of Experimental Medicine and Biotechnology, Post Graduate Institute of Medical Education and Research (PGIMER), Chandigarh, India

^5^Department of Histopathology, Post Graduate Institute of Medical Education and Research (PGIMER), Chandigarh, India.

^6^School of Public Health, Post Graduate Institute of Medical Education and Research (PGIMER), Chandigarh, India.

**
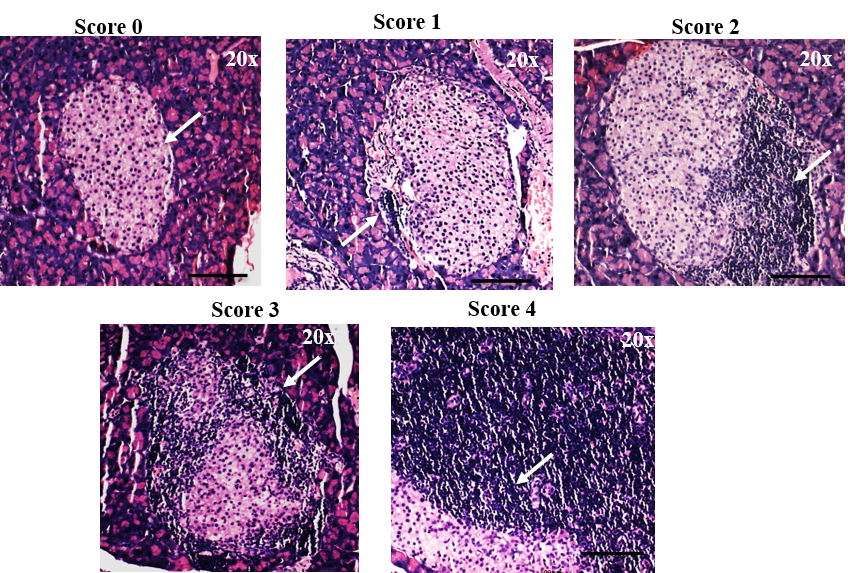
**

**Fig S1: Insulitis scoring of pancreas sections.** Representative histological sections of pancreas from NOD mice showing different insulitis scores. Score 0, no insulitis; Score 1, peri-insulitis; Score 2, mild insulitis (<25% infiltration); Score 3, severe insulitis (25% to 75% infiltration) and Score 4, destructive insulitis (> 75% infiltration). Arrows shows region of infiltration. Images were acquired at 20x magnification, scale bars indicate 200 µm.


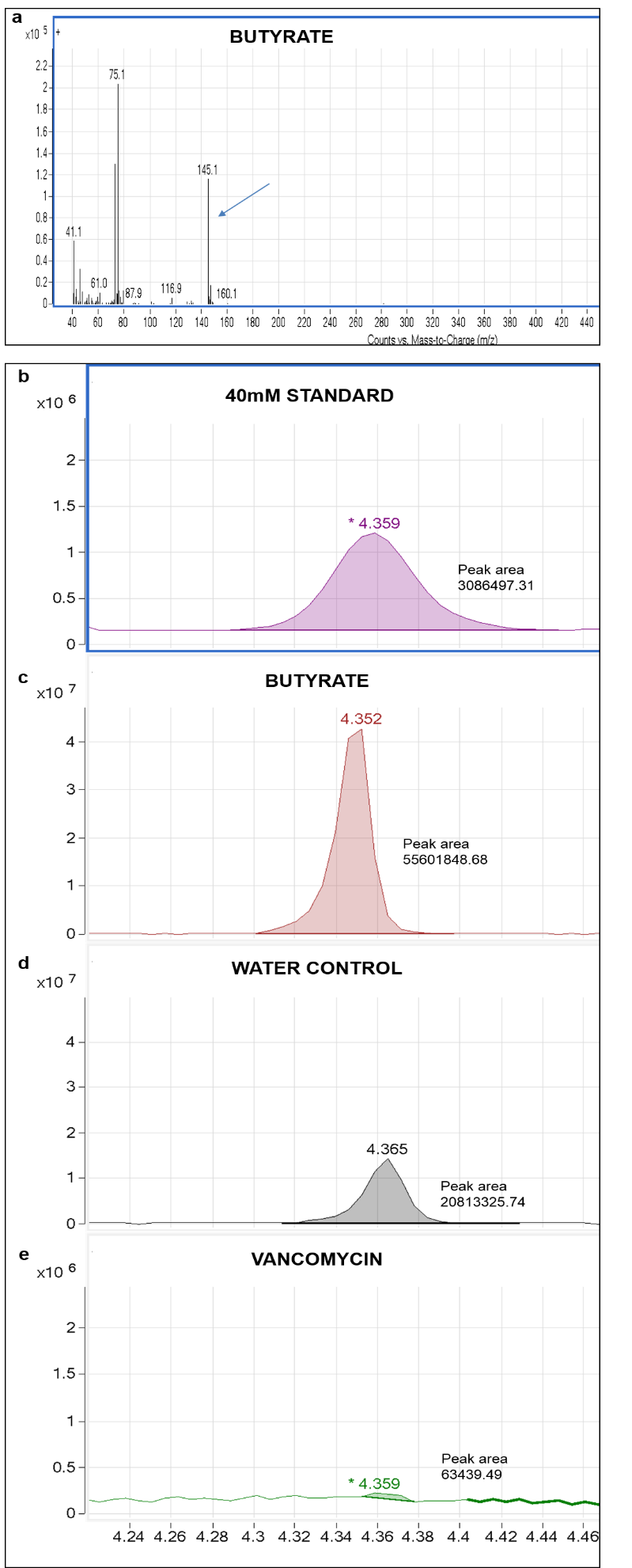


**Fig S2: GC-MS analysis of colonic contents of butyrate treated mice**

**(a)** Graph showing count versus M/z ratio of butyric acid (145.1) in butyrate treated mice. NOD female mice were treated with sodium butyrate (150mM) or water (control mice). A set of mice (n=3) were also treated with vancomycin (500mg/L) in drinking water for 4 weeks. The fluid intake was monitored and the water was changed every 3 days (1). Colonic contents were assessed by gas chromatography–mass spectrometry (GC–MS) as previously described (Hoving et. al 2018). Briefly, 10mg of colonic content was homogenized by vortexing and centrifuged (1400×g, 10 min.) (2). The metabolite from aqueous layer was extracted by oxymation process involving addition of 500uL methoxamine hydrochloride and incubation for 30 min at 60⁰C followed by addition of NaCl and HCl. Ethyl acetate was added to separate the organic phase. The extract was derivatized by adding 100µl BSTFA, 100µl acetonitrile and 10µl pyridine. The vials were tightly sealed, and heated at 80^ο^C for 1hr. The derivatized samples were run on GC system [(7890B), MSD (5977A)] Agilent Technologies (Santa Clara, CA). Separation was performed using HP-5MS column (30m×0.25mm×0.25µm). The sample injection volume was 1.0 µl with a split ratio of 10:1. The flow rate of the carrier gas (high purity Helium) was 14.484 ml/min. GC oven temperature program consisted of 60^ο^C for 4 min, after which temperature was ramped to 200⁰C at 10⁰C per min and held for 2 min. Mass spectra were acquired in range of 50-600 mass at normal scanning mode. Agilent MASS HUNTER software was used to check the presence of butyric acid followed by confirmation from NIST library.

**(b-e)** GC chromatograms showing retention time and peak area under curve (AUC) of butyric acid in, b) Standard sample (40mM butyric Acid), c) Colonic extract of sodium butyrate treated mice, d) Colonic extract of control (untreated) mice, e) Colonic extract of vancomycin treated mice receiving plain drinking water.


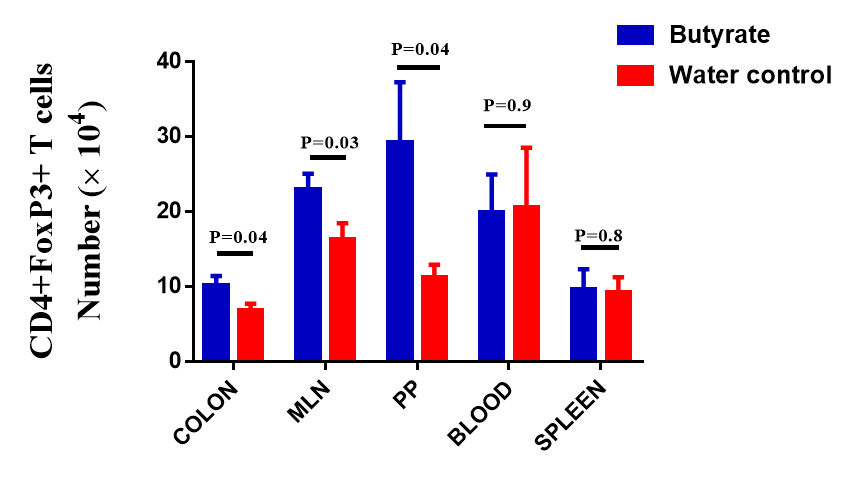


**Fig S3: Butyrate treatment induces Tregs in the colon and the GALT.** Bar diagrams depict (mean±SEM) absolute counts of CD4+FoxP3+ Tregs in the colon (12-13), MLN (n=10), PP (n=4-6), blood (n=6-11) and spleen (n=5-7) of butyrate-treated and water-control hyperglycemic NOD female mice, post 6 weeks of treatment.

**
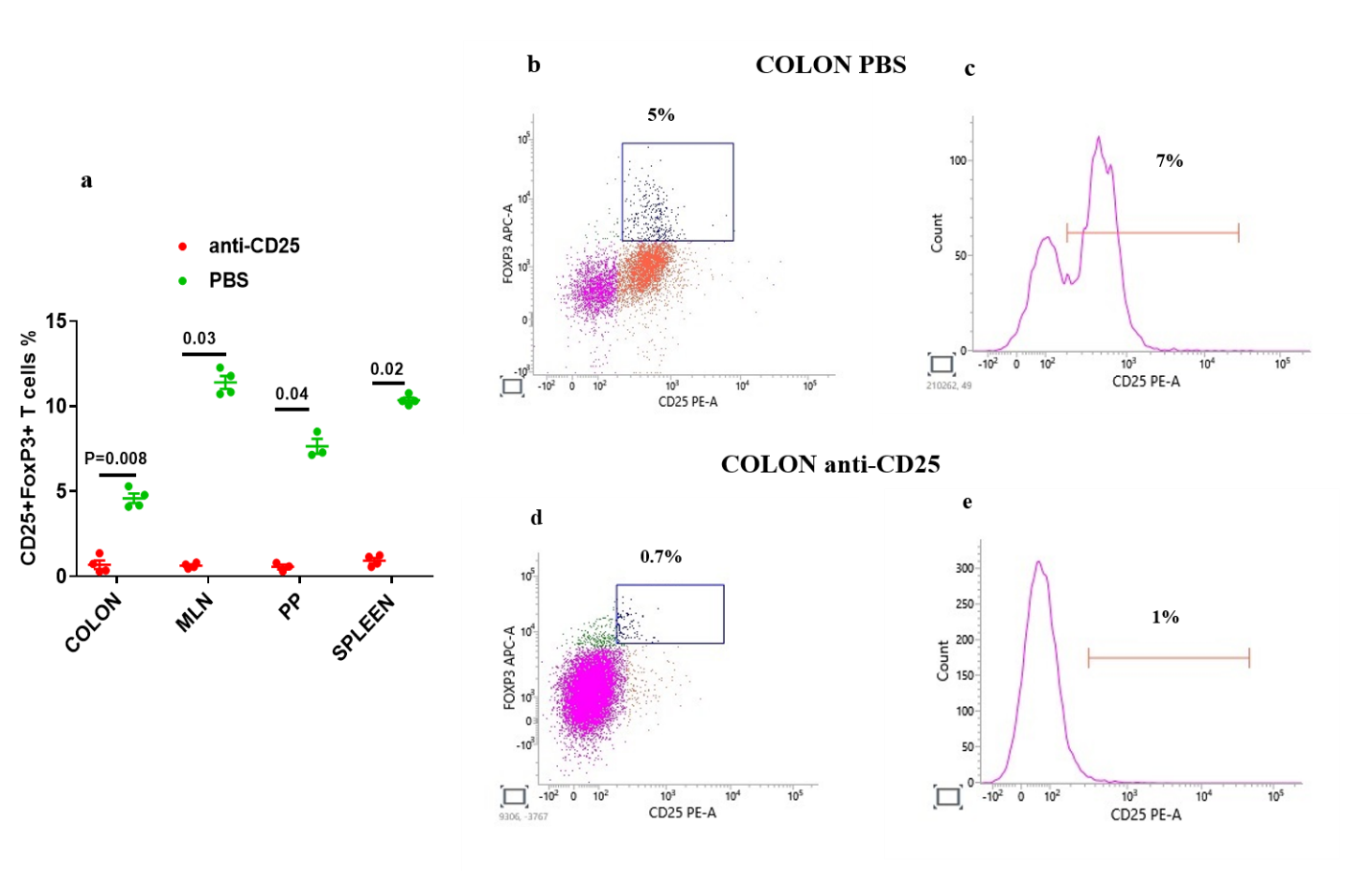
**

**Fig S4: Abolishment of protective effect of butyrate upon depletion of Tregs**. Hyperglycemic NOD female mice were treated with sodium butyrate for 6 weeks following which two intraperitoneal injections of anti-CD25 antibody (0.5mg in PBS) (treatment group, n=4) or PBS alone (control group, n=4) were given one week apart. a) Frequency of CD25+FoxP3+ Tregs (%) in the colon, MLN, PP and spleen (n=4). Representative flow cytometry plots showing the frequency of CD25+FoxP3+ T cells in the colon of, b) PBS treated and, d) anti-CD25 treated NOD mice. Representative flow cytometry histogram plots comparing CD25 expression in CD3+CD4+ T cells isolated from colon of, c) PBS treated and ,e) anti-CD25 treated NOD mice. Data are shown as mean ± SEM. Statistical significance was determined by Mann-Whitney U test, with P < 0.05 considered significant.


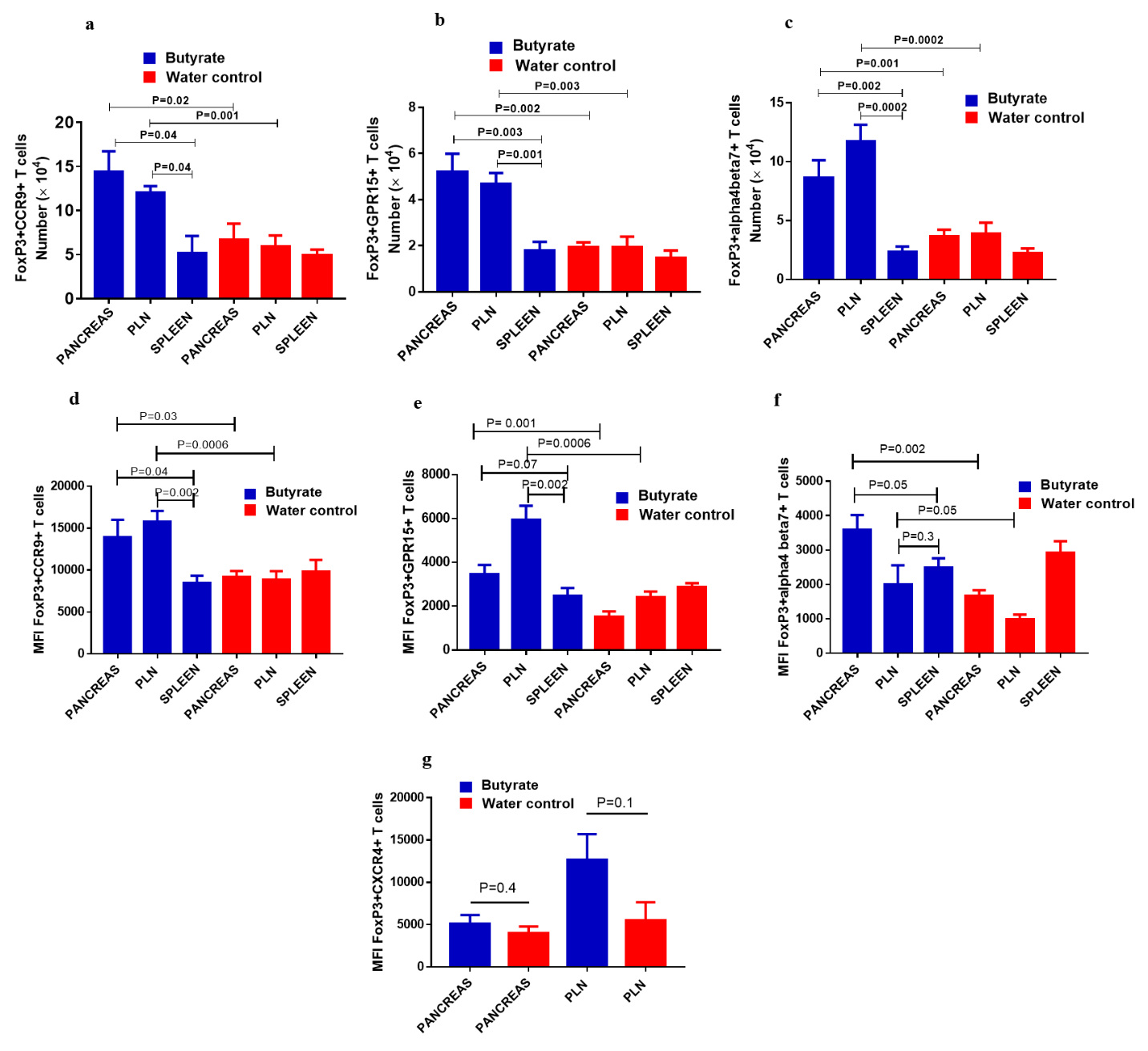


**Fig S5: Butyrate treatment promotes the accumulation of gut homing receptor expressing Tregs in the PLN and pancreas**. Bar diagrams depict absolute counts of, a) FoxP3+CCR9+ Tregs, b) FoxP3+GPR15+ Tregs and c) FoxP3+α4β7+ Tregs in pancreas (n=7), PLN (n=7), and spleen (n=7). MFI of d) CCR9, e) GPR15 and, f) α4β7 on FoxP3+Tregs in pancreas (n=7), PLN (n=7), and spleen (n=7). g) MFI of CXCR4 in FoxP3+ Tregs in pancreas (n=5-6) and PLN (n=6-7). Results are displayed as Mean±SEM. Statistical significance was determined by Mann-Whitney U test, with P < 0.05 considered significant.


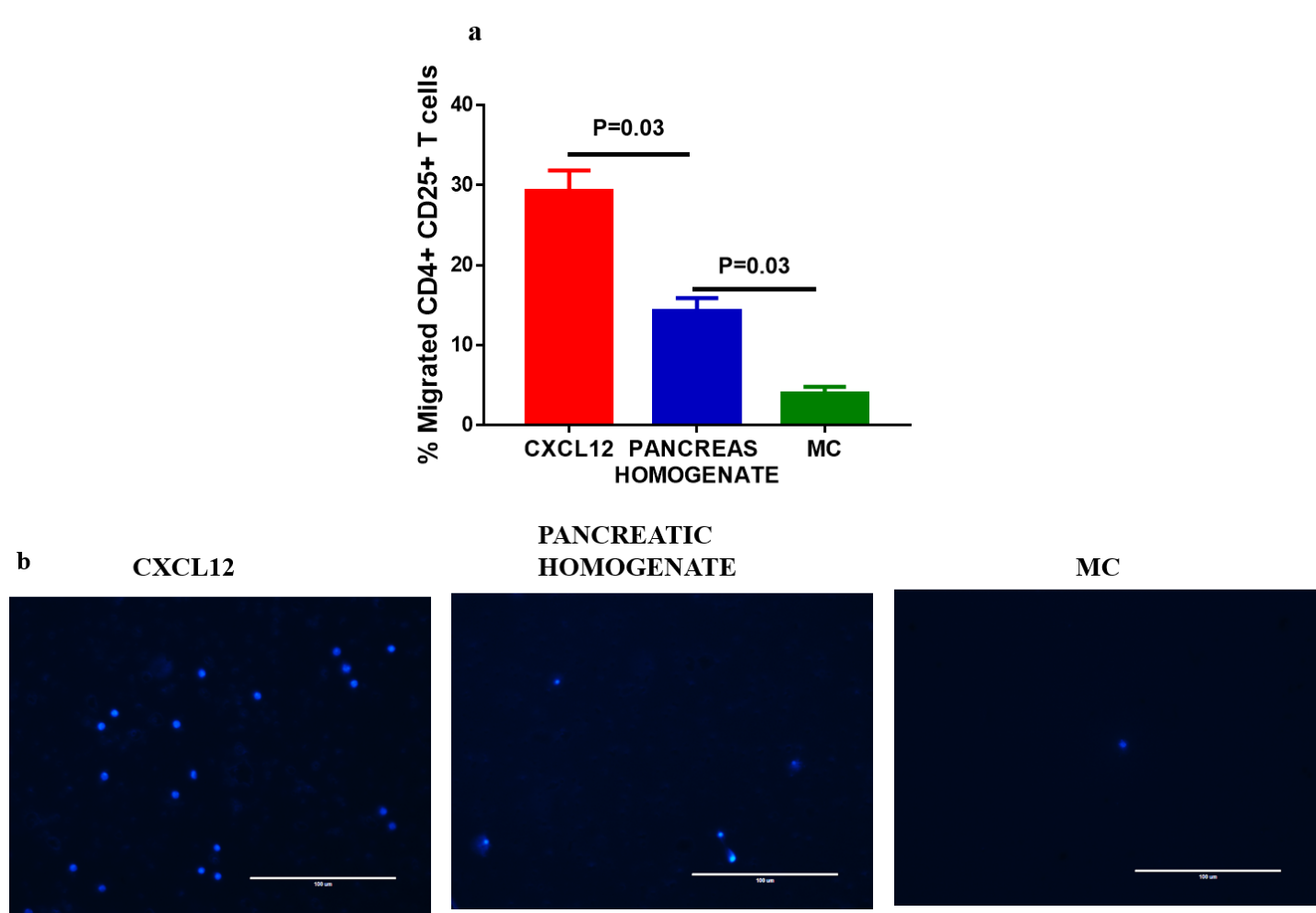


**Fig S6: Butyrate induced colonic Tregs show chemotaxis towards pancreas**

CD4+CD25+ regulatory T cells were isolated from the colon of butyrate treated mice and 1.5 x 10^5^ cells were added to the upper well of a transwell chamber. After 24 h of incubation, the cells in the lower well were collected, stained for CD4 and CD25 and analyzed by flow cytometry. a) Frequency of CD4+CD25+ T cells migrating toward CXCL12, pancreatic homogenate and media control (MC). b) Photomicrographs of DAPI labelled Tregs migrating towards CXCL12, pancreatic homogenate and MC, respectively. The slides were viewed on the fluorescence microscope and images were visualized using Pearlscope software at 40x magnification; scale bars indicate 100 µm.


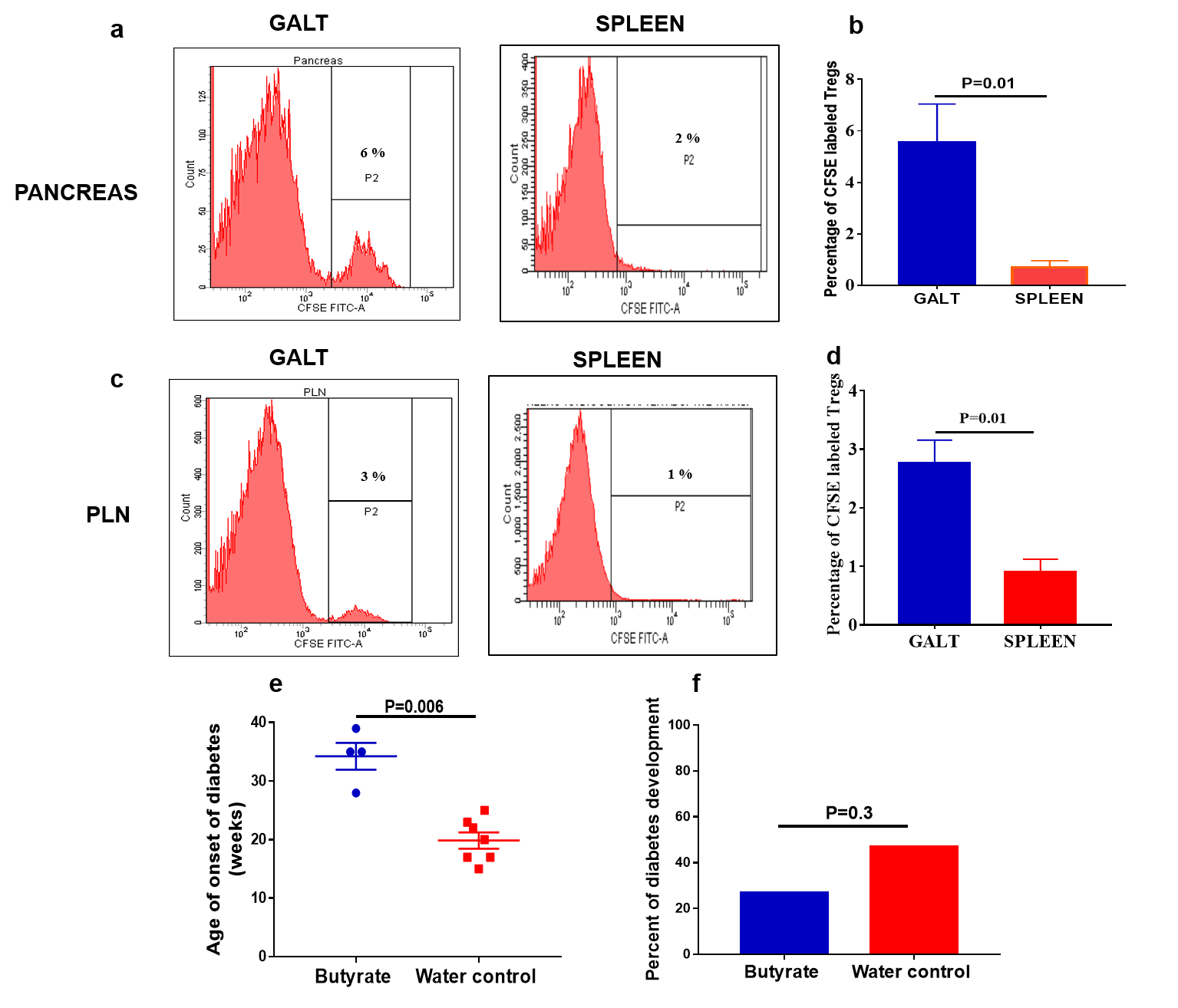


**Fig S7:** **Migration of CFSE-labelled Tregs isolated from the GALT and spleen (control) of butyrate treated mice**. Representative histograms showing the frequency of CFSE labelled Tregs in the (a) Pancreas and (c) PLNs of recipient NOD mice after 5 days of adoptive transfer. Percentage of CFSE Tregs was analyzed by flow cytometry from (b) Pancreas (n=6) and (d) PLN (n=6) following adoptive transfer of Tregs isolated from GALT and spleen, respectively. e) Delay in the onset of diabetes after adoptive transfer of Tregs from butyrate treated mice (n=15) and water control group (n=15) in 6-8 weeks old NOD mice. f) Frequency of diabetes incidence in butyrate and water control groups until 40 weeks. Results are displayed as Mean±SEM. Statistical significance was determined by Mann-Whitney U test, with P < 0.05 considered significant.


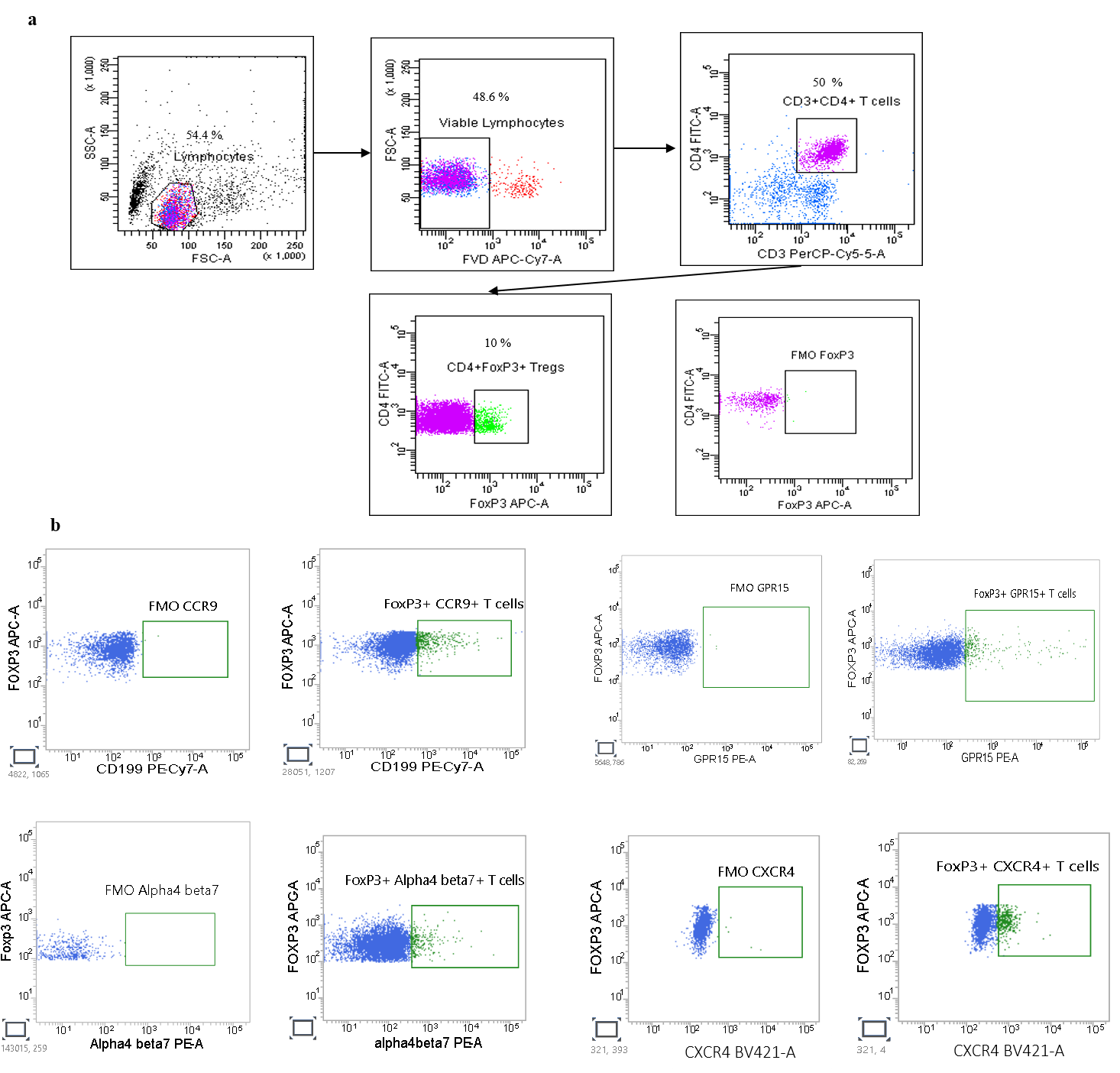


**Fig S8: Gating strategy of Tregs and homing receptors.** a) Representative gating strategy of flow cytometric analysis of Tregs from mouse mesenteric lymph nodes (MLN). Single cell suspension was obtained from MLN of NOD mice lymphocytes. They were stained with fixable viability dye (Zombie NIR Fixable Viability Kit) to gate the viable cells. The cells were stained with fluorescent conjugated antibodies CD3(PerCP), CD4 (FITC), FoxP3 (APC) based on surface or intranuclear staining protocols respectively. Data were acquired on BD FACS Canto II flow cytometer and analyzed with BD FACS DIVA software Lymphocytes were identified by their scatter properties (SSC-A×FSC-A plots).Fluorescent minus one (FMO) tubes were used as controls to set the gates.

b) Representative gating strategy of flow cytometric analysis of gut homing receptors and CXCR4 on Tregs. Following the gating of Tregs (CD4+FoxP3+ T cells), FoxP3+CCR9+T cells, FoxP3+GPR15+ T cells, FoxP3+alpha4 beta7+ T cells, FoxP3+CXCR4+ T cells were gated. Fluorescent minus one (FMO) tubes were used as controls to set the gates for each receptor.

**Table S1: Consumption of water (mL) with and without butyrate by hyperglycemic NOD mice**

| Residual volume in water bottle over time (mL) | | | | | | | | |
| --- | --- | --- | --- | --- | --- | --- | --- | --- |
| Treatment Group | Day 0 (input) | Day 3 | Day 6 | Day 9 | Day 12 | Day 15 | Day 18 | Day 21 |
| Butyrate (150 mM) | 200 | 150 | 140 | 130 | 120 | 100 | 80 | 80 |
| Water control | 200 | 140 | 130 | 120 | 100 | 80 | 80 | 70 |

**Water was replaced every 3 days and 3 mice were kept per cage**

**Average consumption of water (ml) per mice**

| Treatment Group | Day 3 | Day 6 | Day 9 | Day 12 | Day 15 | Day 18 | Day 21 |
| --- | --- | --- | --- | --- | --- | --- | --- |
| Butyrate (150 mM) | 17 | 20 | 23 | 26 | 34 | 40 | 40 |
| Water control | 20 | 23 | 27 | 33 | 40 | 40 | 43 |

**Table S2: Assay ID’s of various chemokines used for qRT-PCR**

| **Gene** | **Assay ID** |
| --- | --- |
| CXCL10 | Mm00445235_m1 |
| CCL19 | Mm00839967_g1 |
| CCL22 | Mm00436439_m1 |
| Madcam1 | Mm00522088_m1 |
| CXCL12 | Mm00445553_m1 |
| CCL5 | Mm01302427_m1 |
| CCL4 | Mm99999221_m1 |
| HPRT | Mm00446968_m1 |

**References**

1. Smith PM, Howitt MR, Panikov N, Michaud M, Gallini CA, Bohlooly-y M, et al. (2013)The microbial metabolites, short-chain fatty acids, regulate colonic Treg cell homeostasis. Science;341(6145):569-73.

2. Hoving LR, Heijink M, van Harmelen V, van Dijk KW, Giera M. GC-MS analysis of short-chain fatty acids in feces, cecum content, and blood samples. Clinical Metabolomics: Springer; 2018. p. 247-56.
